# A digital memories based user authentication scheme with privacy preservation

**DOI:** 10.1371/journal.pone.0186925

**Published:** 2017-11-30

**Authors:** JunLiang Liu, Qiuyun Lyu, Qiuhua Wang, Xiangxiang Yu

**Affiliations:** 1 School of Cyberspace Security, Hangzhou Dianzi University, Hangzhou, ZheJiang, China; 2 School of Computer Science and Technology, Hangzhou Dianzi University, Hangzhou, ZheJiang, China; University of Texas at San Antonio, UNITED STATES

## Abstract

The traditional username/password or PIN based authentication scheme, which still remains the most popular form of authentication, has been proved insecure, unmemorable and vulnerable to guessing, dictionary attack, key-logger, shoulder-surfing and social engineering. Based on this, a large number of new alternative methods have recently been proposed. However, most of them rely on users being able to accurately recall complex and unmemorable information or using extra hardware (such as a USB Key), which makes authentication more difficult and confusing. In this paper, we propose a Digital Memories based user authentication scheme adopting homomorphic encryption and a public key encryption design which can protect users’ privacy effectively, prevent tracking and provide multi-level security in an Internet & IoT environment. Also, we prove the superior reliability and security of our scheme compared to other schemes and present a performance analysis and promising evaluation results.

## Introduction

With the rapid growth of the Internet and the Internet of Things (IoT), a great number of personalized services are embedded in our daily lives, with many and increasing interactions with our personal devices. Unfortunately, many of the existing authentication mechanisms are considered weak, insecure or outdated, and these problems will continue to hamper the development of cyber space until a new, alternative method is developed.

Traditional knowledge-factor based authentication methods, for example, the text password, are based on letters, characters and numbers, which have long been considered insecure and vulnerable to guessing, dictionary attack, key-logger, shoulder-surfing and social engineering [1–5]. The fact users tend to choose very easy memorable passwords means they are often easily guessed. Worse still, a user has 26 online accounts on average [6,7], with each account demanding the user to set a password for logging in, so it’s really unsurprising that they would use the same password for all these online accounts, even some important online accounts, such as for online banking. Thus, possession-factor based authentication methods, such as ID cards [8], smartphones or one-time password (OTP) generators are used as an additional authentication scheme in some important online account authentications [9]. The disadvantage of these schemes is that users have to carry these devices all the time, and some of this hardware is difficult or complex to use for some users. They are also vulnerable to man-in-the middle attacks [2,3,5,9].

Considering the weakness of knowledge-factor based authentication methods and the complexity of possession-factor based authentication methods, biometric-factor based authentication methods have been introduced. These schemes rely upon unique inheritance factors such as finger prints, iris etc., which are unchanging during the lifetime of a human to achieve more security than the traditional schemes. But the major problem of adopting biometric-factor based authentication schemes is the high cost of additional devices needed for the identification process [10,11,12,13].

Graphical passwords have been a popular topic, and many practical ideas have been developed, like Passfaces™ [14] and Passpoint™ [15,16]. These systems require users to choose photos provided by the system as an authentication mechanisms. Nowadays, researchers have found that compared with static photos, it is easier for Users to recall their passwords by using their digital memories [7,17], and with the increasing number of devices within the Internet and IoT, Users’ digital memories are more rich, colorful and available to be used as authentication mechanisms.

Based on this, Shone et al. [18] proposed a scheme named *Digital Memories Based Mobile User Authentication for I0T*, which required users to upload their digital memories and use them as authentication materials in IoT environments. However, there are imperfections in their scheme which is vulnerable to privacy disclosure attack and user tracking attack. In this paper, we enhanced Shone et al*’s* [18] scheme to provide privacy preservation, anti-tracking and a multi-level security scheme for easing user authentication. In our scheme, we adopt homomorphic encryption to protect users’ digital memories as well as users’ privacy effectively, and apply public key encryption to improve data transmission security.

The rest of the paper is organized as follows: Section 2 provides background information and existing research on authentication and digital memories, and an analysis of Shone et al*’s* [15] scheme. We propose our scheme in detail in Section 3. In Section 4 we test the security of our protocol and present our evaluation result. In section 5 we present the potential applications of our scheme and analyze performance. We draw conclusions in Section 6.

## Related works

### Digital memories

Human digital memory was first conceptualized in 1945 by Bush in his Memex system [18]. He described a device which was a place for storing books, records and communications and was envisioned as an “enlarged intimate supplement to memory” [19]. Nowadays, the rapid development of smart and wearable devices has made this idea a reality, and has paved the way for ‘life-logging’. Users can use such devices to record aspects of their lives in digital form [20,21], such as the photos taken with their smartphone. These digital memories presents a highly recognizable identity to specific individuals and are much easier to remember than traditional passwords, so they have the potential to be used for authentication.

### Recognition-based and Recall-based authentication

Recognition-based and Recall-based authentication stem from graphical passwords [3,22], and they are still effective when we apply them to digital memories, including video, audio, text, etc.

In recognition-based systems, a group of digital memories is displayed to the user and an accepted authentication requires correct digital memory objects (video, image, etc.) to be clicked or touched in a particular order. An example of a recognition-based system is Passfaces™ developed by Real User Corporation [14]. In Passfaces™ the user is asked to choose four or more images of human faces from a face database provided by the system to use as their future password. In the authentication stage, the user sees a grid of nine faces, including one face chosen by the user and eight unfamiliar faces. The user recognizes and clicks on the familiar face. This process is repeated several times. The user is authenticated if he or she identifies the four faces they have previously chosen.

In recall-based systems, the user is asked to reproduce digital memories that he or she created or selected earlier during the registration phase. A typical recall-based authentication scheme is Passpoint™ [15,16]. In Passpoint™, the image can be an arbitrary photograph or paintings provided by the system with many clickable regions. The user is required to click on some predefined positions on the image in a particular order to be authenticated.

### Analysis of Shone et al’s scheme

**Fig 1:**
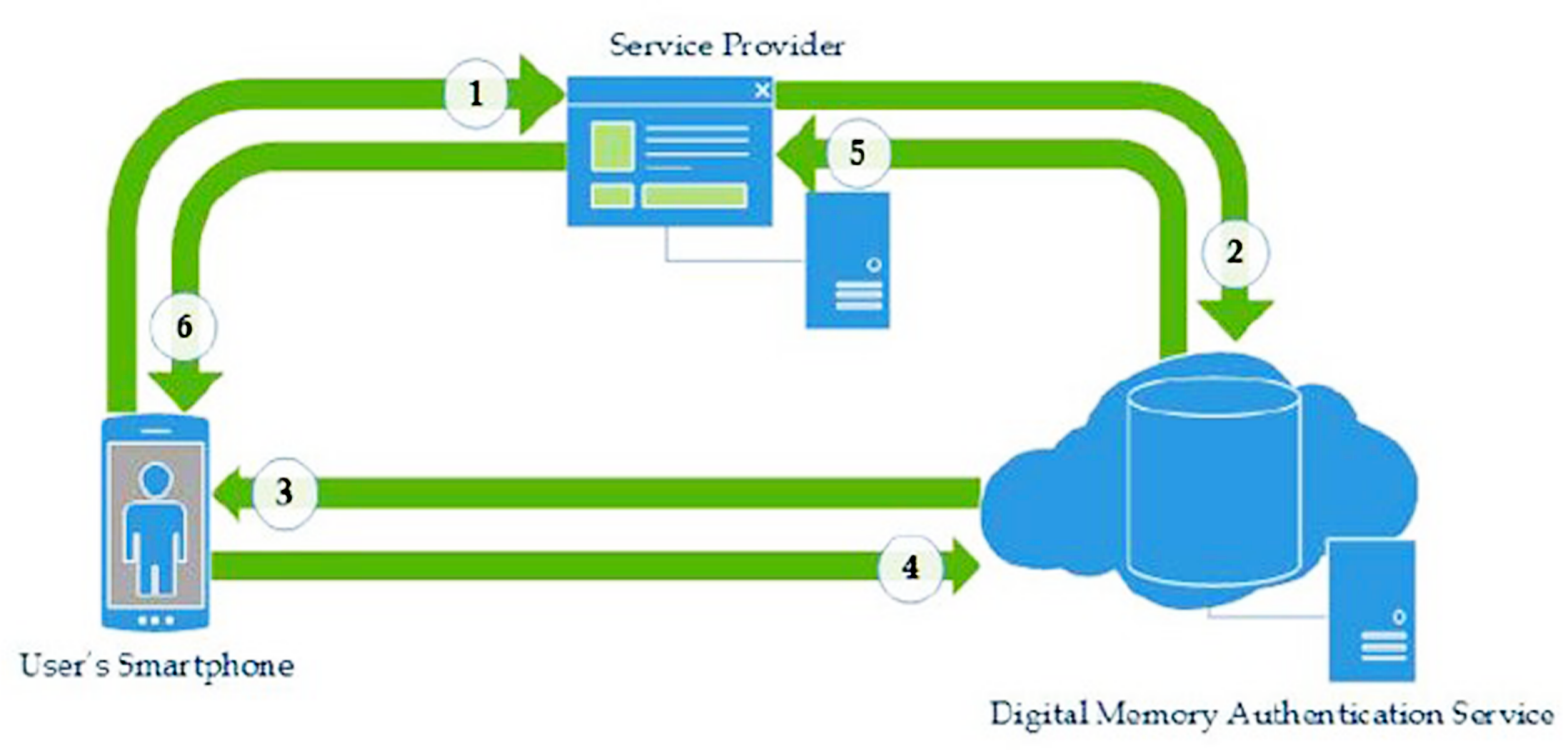
Authentication mechanism overview of Shone et al’s scheme.

Shone et al. [18] proposed a digital memories based user authentication scheme for mobile devices. There are three main actors in their scheme, including the user’s smartphone (US), service provider (SP) and the digital memory authentication service (DMAS). The US serves as an independent platform allowing communications with other actors. As this is a mobile user authentication scheme, the physical presence of the US with the user also becomes an authentication factor. The SP provides a particular service to the user where the user must register their device with the SP. And the DMAS hosts the user’s digital memories in a cloud environment and provides part of the authentication using their memory data. An overview of their scheme is shown in Fig 1.

Shone et al*’s*. [18] scheme follows 6 steps, shown in Fig 1. Symmetric encryption is used to secure communications between the actors, as they all have pre-existing relationships.

**Step 1:** The user requests access to the SP’s service, where the user’s device has been registered. The hardware address of the US is first authenticated according to the SP’s records.

**Step 2:** Once the US is verified, the SP negotiates an authentication session with the DMAS for the user, specifying the comprehensiveness (e.g. number of questions) complexity (e.g. difficulty of the questions) of the desired authentication challenge.

**Step 3:** Upon the user requesting the challenge, it is computed by the DMAS to match the SP’s requirements and is then sent to the user along with a session key (sKey). At this stage, all potentially useful meta-data has been stripped from any digital memory data used. The session key is used as a marker to prevent unauthorized replay attacks, in place of using nonces (arbitrary numbers that may only be used once). Each sKey is time-stamped, has a limited lifespan and is tied to the initiating IP address. A challenge/response mechanism is then used to authenticate the US against the DMAS. These challenges are computed by the DMAS using the user’s digital memories and the SP’s requirements.

**Step 4:** The user sends his response to the challenge back to the DMAS, and the DMAS validates the answers.

**Step 5:** The DMAS returns the authentication challenge result to the SP.

**Step 6:** If the authentication result is successful, the user will be granted access by the SP. And the user and the SP share the same sKey.

This scheme will provide challenges of different complexity in the authentication according to the required verification level; if the level is normal, the DMAS will give a challenge involving arranging several images in an ascending chronological order. As online banking accounts are considered high risk, this would require authentication challenges that match the higher complexity and comprehensiveness expected. For example, the DMAS may give a photo and ask about some details related to it, such as the device used to take the image and the year or month the photo was taken etc.

Although Shone et al*’s*.[18] scheme made the authentication process easy for users as well as hard to attack with intelligence (challenges need user recognition or recall) and uncertainty (each authentication doesn’t have the same answer), there exist some weakness or disadvantages. The main weakness or disadvantages of Shone et al*’s*.[18] scheme are as follows:

- Digital memory objects stored in the DMAS without encryption will introduce potential privacy disclosure risk

The DMAS stores users’ digital memories in a plaintext way; if the DMAS was attacked, there are potential privacy disclosure issues. For example, devices embed photo EXIF information when a user takes photos, and EXIF includes nearly all the details of the photo which are used by Shone et al.[18]’s scheme and are usually privacy-related. Worse still, if the DMAS is semi-trust (rigidly follows the protocol but curious) itself, it would misuse users’ digital memories and may cause property damage to users.

- User’s devID as the key factor of authentication stored in the SP will bring a single point failure attack and a user tracking attacks.

The SP doesn’t provide a service to the user unless the user registers his device (devID) to it. That means the user’s devID will be used and stored in each SP’s database, which means that if any one of the SPs was attacked, the user’s devID can be stolen, forged or misused.And also since the user’s devID isn’t changed, tracking attacks can be launched.

- Authentication process with whole symmetric encryption will result in man-in-the-middle attack. High database load on SP.

In Shone et al*’s*.[18] scheme, they used symmetric encryption to secure communications among the actors, which means before the authentication stage the keys need to be transmitted among the actors, a situation which presents potential risks. This is especially vulnerable to man-in-the-middle attack. Moreover, it increases the load on the actors’ Web servers (the SP and the DMAS) since the servers need to receive, store and transmit the keys for each user when authenticating, which may reduce efficiency and cause a huge waste of network resources.

## Proposed scheme

There are also three main actors in our scheme: The user, the service provider and the DMAS. The authentication process follows four phases: Phase 1: The SP registers to the DMAS; Phase 2: The user registers to the DMAS; Phase 3: The user logs in to the SP; Phase 4: The user logs in to the DMAS to maintain digital memory objects.

For clarity of explanation, notations adopted in this study are summarized in Table 1.

**Table 1:** Notations.

*K*_d_	DMAS’s public key
$K_{d}^{-1}$	DMAS’s private key
*K*_*ud*_	User’s symmetric key
*K*_*u*_	User’s ElGamal public key,{G, q, g, h}
$K_{u}^{-1}$	User’s ElGamal private key,{x}
TS_U	Timestamp from the User
TS_S	Timestamp from the SP
TS_D	Timestamp from the DMAS
Relation info	Related information of group digital media data
IP_U	User’s IP address
DM	User’s digital memory objects
EDMO	Encrypted digital memory objects
Ans	The answers of the challenge
PID	Partner ID

### The SP registers to the DMAS

We can describe the process with the following formulas[23]:

$$\begin{array}{l} SP\to DMAS:\{Application\}\\ DMAS\to SP:\{K_{d},PID\} \end{array}$$

**Fig 2:**
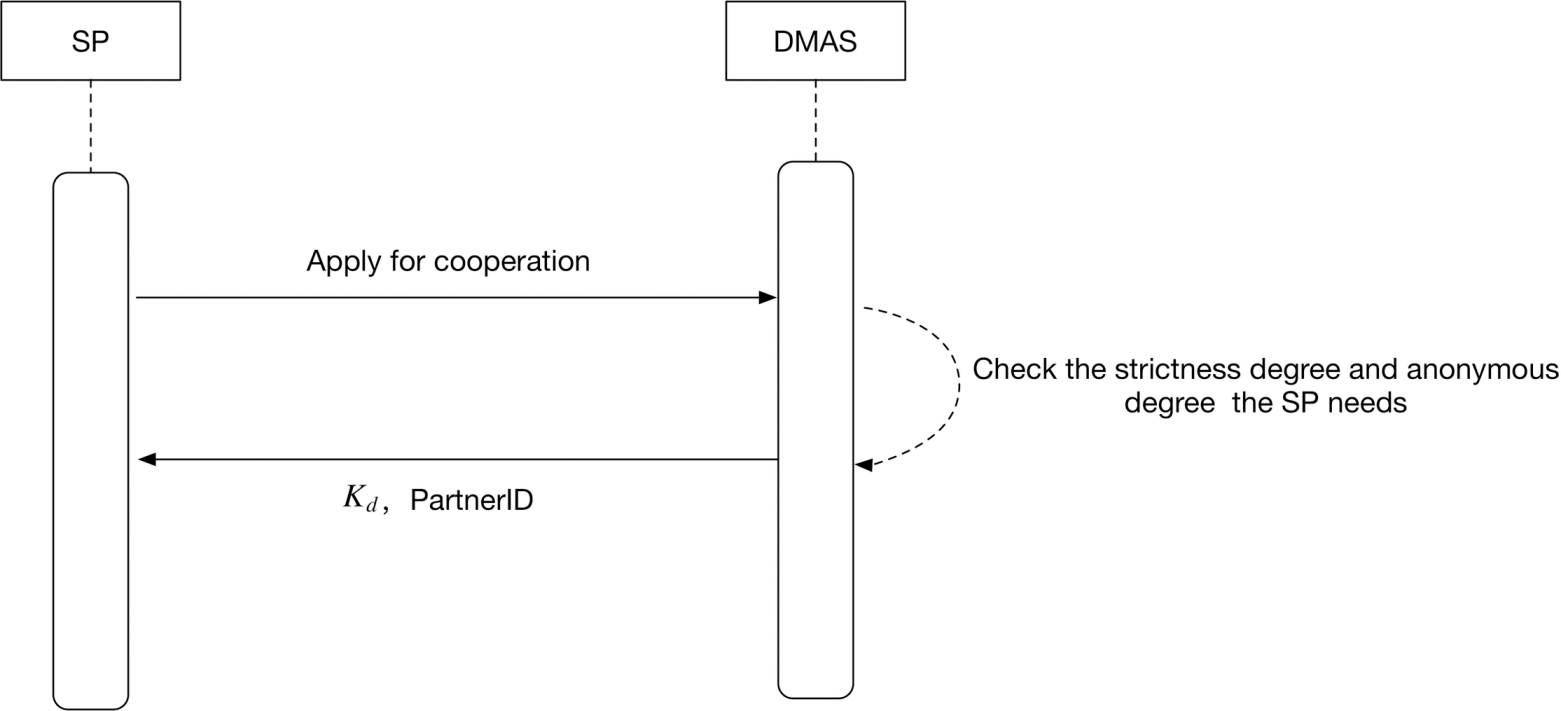
UML sequence diagram of registration protocol between SP and DMAS.

As illustrated in Fig 2, the SP applies to the DMAS for cooperation, and the form of the application can be legal or authentic according to scenarios. The DMAS checks the strictness degree and anonymous degree the SP needs, then gives the SP an exclusive partner ID and distributes its public key *K*_d_. This partner ID will be used when the SP requires authentication for the user. *Ans*' = (*c*_1_,*c*_2_)(*c*_1_,*c*_3_) = (*c*_1_•*c*_1_,*c*_2_•*c*_3_) = (*g*^2*y*^,*m*_1_'•*m*_2_'•*h*^2*y*^).

The partner ID has different levels, which represent different strictness degrees the SP requires. For example, a grid of eight pictures will be displayed for a normal degree partner ID in the authentication phase, while it will increase to twelve pictures for a high degree partner ID, which means more distractors.

Meanwhile, the partner ID also presents the SP’s information requirement degree of the user. If the SP need not know what the user did during his/her previous entry, namely, if the user’s current login has no relevance to his history, the DMAS will give a partner ID of anonymous degree to the SP, which means the user will log in to this SP with anonymous type; all his/her operations in this SP will not be recorded in order to prevent tracking. On the other hand if the SP needs to know the user’s previous information and operations during their entry, such as with online bank and online shopping, the DMAS will give a partner ID of real degree, which means the user will log in to this SP with real type; all his/her operations in this SP will be recorded and bound to this account in order to ensure the user can continue from the previous session.

### The user registers to the DMAS

We can describe the steps with the following formulas:

U→DMAS:{DevID,UserID}KdDMAS→U:{OK}U→DMAS:{{DM}Kud,{Relation_info}Ku,DevID,UserID,TS_U}KdDMAS→U:{h(DevID,UserID,{Relation_info}Ku),IP_U,TS_U}Kd−1

**Fig 3:**
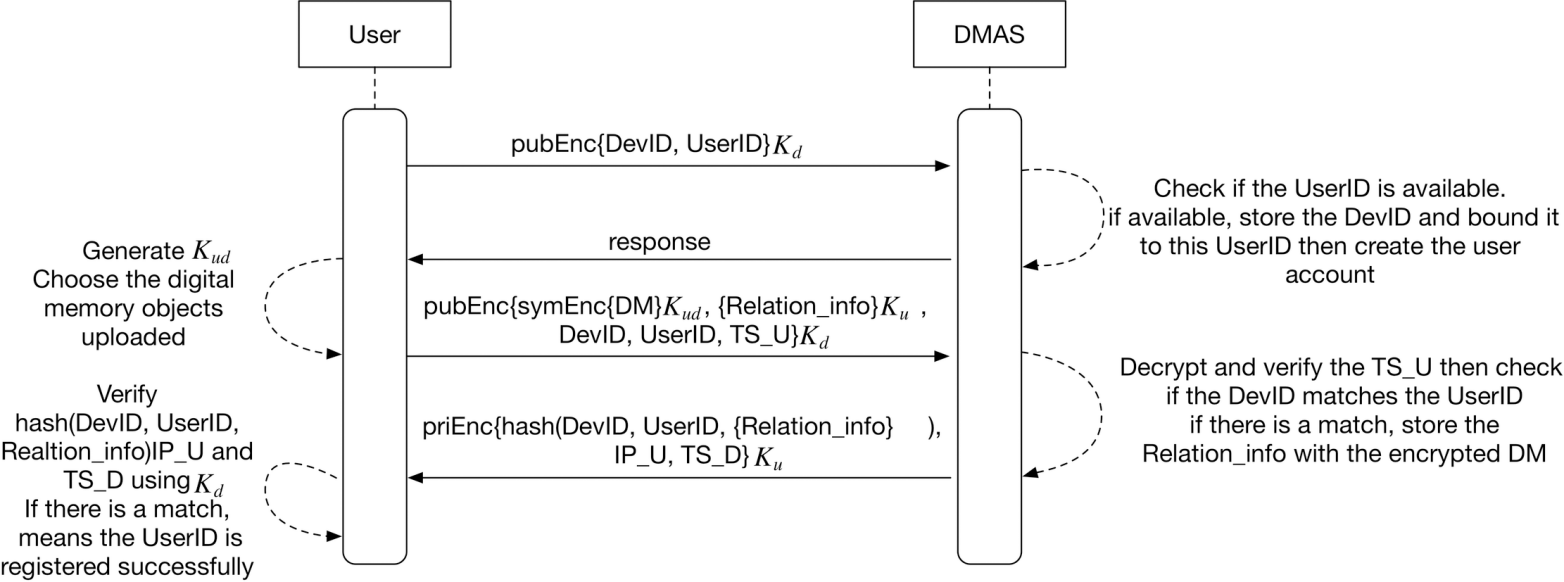
UML sequence diagram of registration protocol between user and DMAS.

The user gets the public key of the DMAS, and then executes the following five steps to finish registering, as illustrated in Fig 3.

**Step 1:** The user randomly chooses a UserID for his/her DMAS account, and generates the unique devID, then he sends UserID and DevID encrypted with the DMAS’s public key *K*_d_. To ensure the uniqueness of devID, we first fetch the device’s motherboard serial number, CPU serial number, hard disk serial number and other unique numbers on the hardware. We then combine them and use the SHA256 algorithm to generate a message digest to be used as the devID of this device.

**Step 2:** The DMAS checks if the UserID is available. If available, the DMAS stores the devID and bounds it to the UserID and takes it as the first factor, on the hardware level, in the authentication stage. Then the DMAS creates the account, and sends the DMAS’s response to the user.

**Step 3:** The user generates a symmetric key *K*_*ud*_ and ElGamal [24] key pair *K*_*u*_, $K_{u}^{-1}$, then encrypts and uploads the encrypted digital memories with related information.

The ElGamal key pair generation process is as follows:

a The user generates an efficient description of a cyclic group G of order q with generator g. See below for a discussion of the required properties of this group.
b The user chooses an x randomly from {1, …… q—1}, and computes *h* ≔ *g*^*x*^
c The user publishes h, along with the description of G, q, g, as his/her public key *K*_*u*_. The user retains x as his/her private key $K_{u}^{-1}$, which must be kept secret. So the symmetric key *K*_*ud*_ and ElGamal private key $K_{u}^{-1}$ will be kept and owned by the user only.

Then the user divides his/her digital memories which will be uploaded into several groups in which any digital memory objects can be included in many different groups on their device. The user then gives a name they like (even a code word or metaphor) to every group and encrypts the digital memory objects chosen with the symmetric key. The user then uploads the encrypted digital memories. While uploading, the DMAS will generate a feature code for every encrypted digital memory object and relate it to its groups. For example, the user creates a group named “food”, and selects five digital memory objects for this group, then, while uploading, the five digital memory objects’ feature codes will be encrypted separately and inserted into a database table whose name is the encrypted outcome of “food”. If other groups want to include these five digital memory objects, they will get a copy feature code of the digital memory object and insert into their database tables. We call these feature codes and group information “related information”. The related information’s database data is all encrypted with ElGamal public key *K*_*u*_ and is then named “relation_info” and sent with UserID and DevID, which are encrypted with the DMAS’s public key *K*_*d*_ before being transmitted.

The details of the process to encrypt related information group data using ElGamal are as follows (we suppose the group name is “food”, and it contains five digital memory objects):

a Use ElGamal public key *K*_*u*_ to encrypt “food”, and use the encrypted outcome as the newly created database table name.
b Use ElGamal public key *K*_*u*_ to encrypt the five digital memory objects’ feature codes separately, the steps are as follows:
  a) The user chooses a random y from {1, …… q—1}, then calculates *c*_1_ ≔ *g*^*y*^ (note: one group’s data shares the same y, and is owned by the user)
  b) The user calculates the shared secret *s* ≔ *h*^*y*^, and maps his/her digital memory objects’ feature code *m*_1_, *m*_2_, *m*_3_, *m*_4_, *m*_5_ onto an element *m*_1_′, *m*_2_′, *m*_3_′, *m*_4_′, *m*_5_′ of G.
  c) The user calculates *c*_2_ ≔ *m*_1_′•*s*, *c*_3_ ≔ *m*_2_′•*s*, *c*_4_ ≔ *m*_3_′•*s*, *c*_5_ ≔ *m*_4_′•*s*, *c*_6_ ≔ *m*_5_′•*s*, and computes the ciphertext (*c*_1_,*c*_2_) = (*g*^*y*^,*m*_1_′•*h*^*y*^), (*c*_1_,*c*_3_) = (*g*^*y*^,*m*_2_′•*h*^*y*^), (*c*_1_,*c*_4_) = (*g*^*y*^,*m*_3_′•*h*^*y*^), (*c*_1_,*c*_5_) = (*g*^*y*^,*m*_4_′•*h*^*y*^), (*c*_1_,*c*_6_) = (*g*^*y*^,*m*_5_′•*h*^*y*^)
c Separately insert the outcome into the database table whose name is the encrypted outcome of “food”.

**Step 4:** The DMAS decrypts and verifies the timestamp and checks if the DevID matches the UserID. If there is a match, the DMAS stores the encrypted relation information with the encrypted digital memories. Then the DMAS will send a data packet which contains the hash values of devID, UserID and the encrypted related information ({Relation_info} *K*_*u*_). In addition, it also contains the user’s IP address (IP_U) and the timestamp from the DMAS (TS_D). All this data is encrypted with the DMAS’s private key $K_{d}^{-1}$.

**Step 5:** The user gets the response and verifies the data. If there is a match, it means the UserID is registered successfully.

### The user logs in to the SP

We can describe the steps with the following formulas:

$$\begin{array}{l} SP\to DMAS:\{PID,TS\_S\}K_{d}\\ DMAS\to U:\{OK\}\\ U\to DMAS:\{DevID,UserID,TS\_D\}K_{d}\\ DMAS\to U:\{EDMO,h(UserID),TS\_D\}K_{d}^{-1}\\ U\to DMAS:\{DevID,UserID,Ans,TS\_U\}K_{d}\\ DMAS\to U:\{h(UserID),Pass\_flag,TS\_D\}K_{d}^{-1}\\ DMAS\to SP:\{h(PID),UserID/AnonymousID,TS\_D\}K_{d}^{-1}\\ SP\to U:\{OK\} \end{array}$$

**Fig 4:**
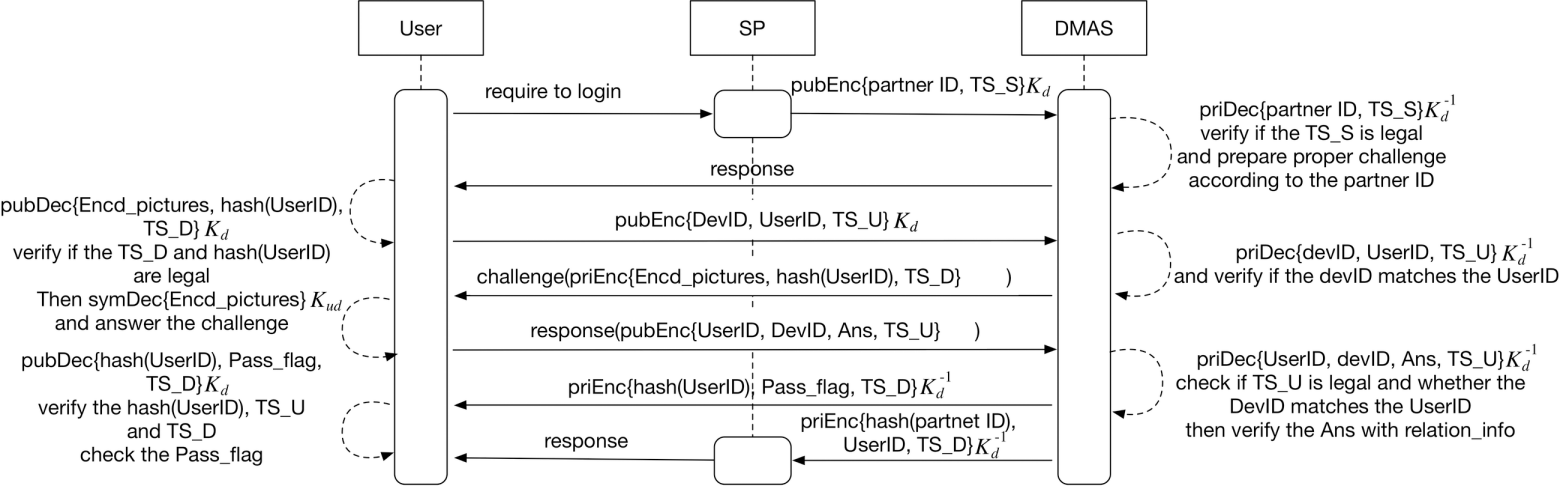
UML sequence diagram of login protocol between the user and the SP.

When the user wants to get service from the SP, he should be authenticated by the DMAS, the the login phase has 7 steps, as illustrated in Fig 4:

**Step 1:** When the user wants to log in to the SP, the SP will require DMAS for authentication. The SP encrypts it’s partner ID and the timestamp from the SP (TS_S) with *K*_*d*_ and sends them along with the user IP address to the DMAS.

**Step 2:** The DMAS decrypts the data and verifies if the TS_S is legal, then retrieves the strictness degree (number of digital memory objects) and anonymous degree (anonymous or not) for the desired authentication challenge according to the SP’s partner ID, and redirects the DMAS’s authentication page to the user.

**Step 3:** Upon receiving it, the user inputs his/her UserID of the DMAS account, computes the devID and then encrypts the UserID, the devID and the timestamp (TS_U) with *K*_*d*_. Finally, the user sends them to the DMAS.

**Step 4:** The DMAS decrypts the data with $K_{d}^{-1}$ and verifies if the devID matches the UserID. Then the DMAS, according to strictness degree and anonymous degree of the SP (see step 2), sends the challenge to the user which contains encrypted digital memory objects (EDMO) and encrypted group names for authentication, the hash value of UserID and the timestamp from the DMAS (TS_D). All this data is encrypted with $K_{d}^{-1}$.

**Step 5:** The user decrypts the data with *K*_*d*_ and verifies if the TS_D and hash value (UserID) are legal, then decrypts the encrypted digital memory objects and group names with *K*_*ud*_, $K_{u}^{-1}$ and answers the challenge. After finishing the answers, the user submits the response which contains the UserID, the devID, the answers of the challenge (Ans) and the timestamp from the user, this time (TS_U). All of this data is encrypted with *K*_*d*_ too. The Ans is made up of the feature code of the digital memory objects selected by the user, and is encrypted with the user’s public key *K*_*u*_.

**Step 6:** The DMAS decrypts the data and checks if the TS_U is legal and whether the devID matches the UserID. If all is matching, the DMAS then verifies the Ans. Because the Ans is encrypted with the user’s *K*_*u*_, the DMAS needs to verify it with relation_info using Elgamal homomorphic encryption.

The details of the DMAS verifying the Ans with Elgamal homomorphic encryption[25] are as follows:

a After the user gets the group name with $K_{u}^{-1}$, he chooses the right digital memory objects and generates their feature codes, then retrieves the parameter y, and carries out the encryption process. For example, with two digital memory objects, we do the following:
  First, map digital memory objects’ feature codes *m*_1_, *m*_2_ onto an element *m*_1_′, *m*_2_′ of G.
  Second, calculate *g*^*y*^, *h* = *g*^*y*^, the shared secret *s* ≔ *h*^*y*^, and calculate
  *m*_1_′•*s* = *m*_1_′•*h*^*y*^, *m*_2_′•*s* = *m*_2_′•*h*^*y*^,
  Third, construct *Ans* = (*g*^*y*^•*g*^*y*^,*m*_1_′•*h*^*y*^•*m*_2_′•*h*^*y*^) = (*g*^2*y*^,*m*_1_′•*m*_2_′•*h*^2*y*^)
  When DMAS receives the Ans from the user, he chooses the encrypted feature codes (*c*_1_,*c*_2_) of *m*_1_, (*c*_1_,*c*_3_) of *m*_2_, then computes
  *Ans*′ = (*c*_1_,*c*_2_)(*c*_1_,*c*_3_) = (*c*_1_•*c*_1_,*c*_2_•*c*_3_) = (*g*^2*y*^,*m*_1_′•*m*_2_′•*h*^2*y*^), since we have
  (*c*_1_,*c*_2_) = (*g*^*y*^,*m*_1_′•*h*^*y*^), (*c*_1_,*c*_3_) = (*g*^*y*^,*m*_2_′•*h*^*y*^).
b If Ans = Ans’, then the user is authenticated.

Then, the DMAS will give a pass flag to the user which contains the hash value of the UserID, the Pass_flag and the timestamp from the DMAS, this time (TS_D). All this data is encrypted with $K_{d}^{-1}$.

**Step 7:** The user decrypts the data with *K*_*d*_ and verifies the hash value of the UserID and whether the TS_D is legal. If they are legal and the flag is a Pass_flag, it means the login is successful. Meanwhile, the SP will get the response from the DMAS, which contains the hash value of the partner ID. Also, the response contains the UserID (or anonymous ID according to the degree) and the timestamp from the DMAS, (TS_D) this time. All the data in the response is encrypted with $K_{d}^{-1}$. The SP decrypts the data and verifies the TS_D, then gets the UserID and responds to the user. If the degree of this SP is anonymous, the response UserID will be an anonymous ID, which means it has no relation to the real UserID. Otherwise, the response UserID will be the user’s real UserID on the DMAS.

### The user logs in to the DMAS to maintain digital memory objects

When the user needs to update the digital memory objects to achieve a more secure authentication result, he should first launch the login process, then follow step 3 to step 7 of Phase 3. For simplicity, we do not explain this in detail.

And then he follows step 3 and step 4 of Phase 2 to update or maintain his digital memory objects.

## Security analysis & protocol evaluation

In this section, we choose several state-of-the-art schemes which are similar to our scheme to compare the security properties. The comparison can be seen Table 2.

**Table 2:** Security comparison between state-of-the-art schemes and our scheme.

	Digital memory	Multi-level authentication	Privacy leak-proof	Semi-trust SP	User anti-tracking	Encryption method
**PassPoints[16]**	×	√	×	×	×	N/A
**IPAS[10]**	√	√	×	×	×	N/A
**Shone et al ‘s scheme[18]**	√	√	×	×	×	symmetric
**Our scheme**	√	√	√	√	√	Asymmetric

Compared to Passpoints [16], IPAS [10], and Shone et al [18], our scheme has the following advantages:

1. Leak-proof: The DMAS stores users’ encrypted digital memories, and the symmetric key is owned by the user only, which means the DMAS can’t get the user’s clear-text digital memories, which greatly protects the user’s privacy.
2. Free from SP: We change the design of the register dependencies. In our mechanism, the user doesn’t need to have any registration relationship with the SP. The user can log in to the SP as long as he/she has an DMAS account. When the user wants to log in to the SP, the SP will require a DMAS authentication site for authentication. This greatly simplifies the process of the authentication and reduces the possibility of divulging sensitive information, like devID.
3. Anti-tracking: In our scheme, the DMAS checks the anonymous degree the SP needs and gives anonymous ID to the SP who needn’t know the user’s operation history, which prevents unnecessary tracking of the user.
4. Different method: We use asymmetric encryption instead of symmetric encryption to secure the communications among the actors, which means in the authentication stage the session keys do not need to be transmitted among the actors by the network environment, which has potential risks. This will reduce the risk of suffering man-in-the-middle attack. Moreover, it reduces the load on the actors’ Web servers (the SP and the DMAS) because the severs do not need to receive, store and transmit the keys for every user during authentication.

Also, we used the Scyther Tool [26], [27] to provide a formal analysis. Scyther is an automatic security protocol verification tool, which is used to identify potential attacks and vulnerabilities. It has been used to verify numerous security protocols. We used Scyther to evaluate the following properties of our proposed mechanism:

1. Secrecy
2. Replay attack resistance
3. Man-in-the-middle attack resistance
4. Reflection attack resistance

**Fig 5:**
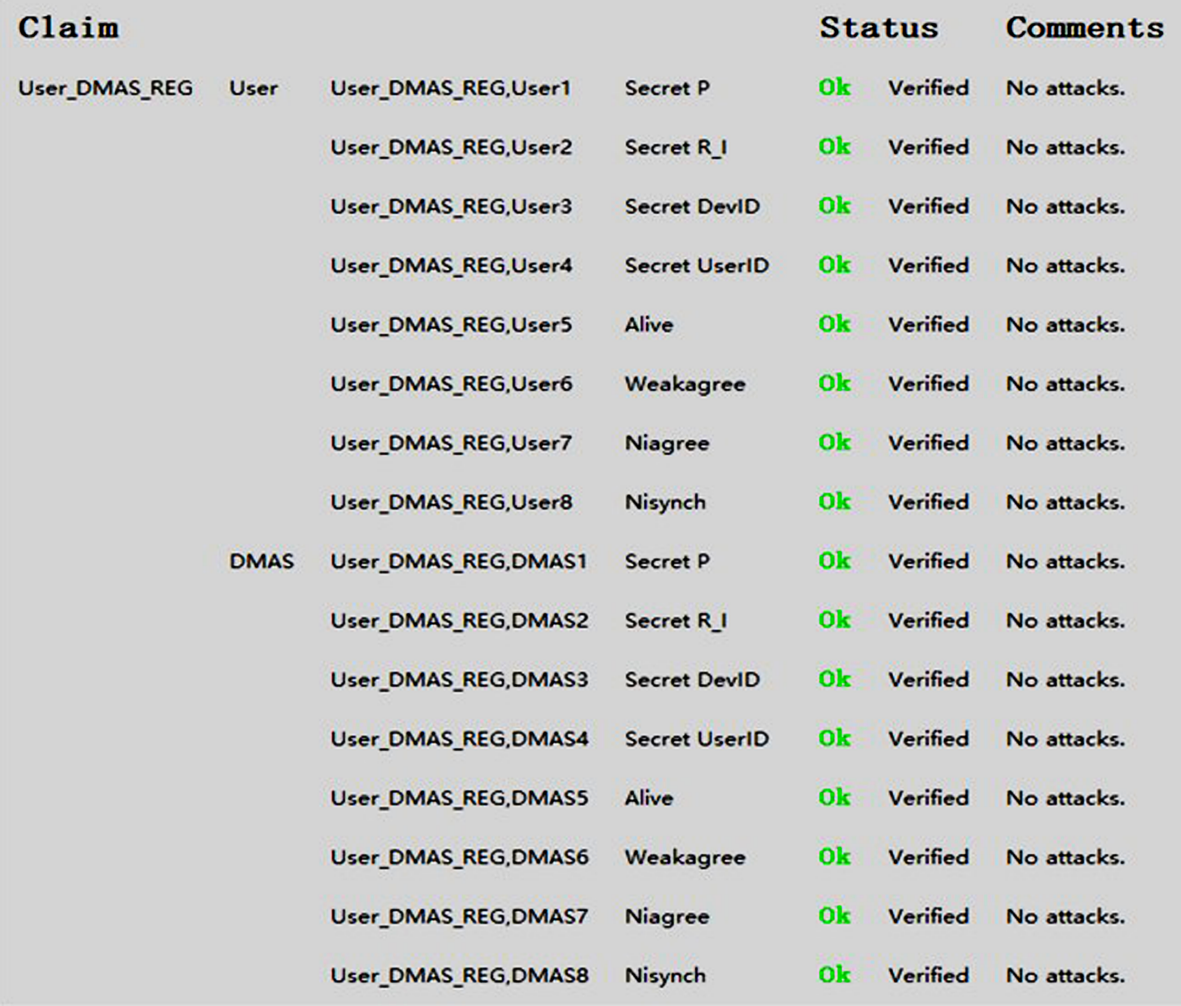
Formal analysis results from Scyther for the the registration protocol. Between the user and the DMAS.

First we used Scyther to analyze the registration protocol between the user and the DMAS whose UML sequence diagram is illustrated in Fig 3. Fig 5 illustrates the results obtained from Scyther’s analysis and we can see there is no attack present in our scheme. The Scyther testing code s in Appendices A.

**Fig 6:**
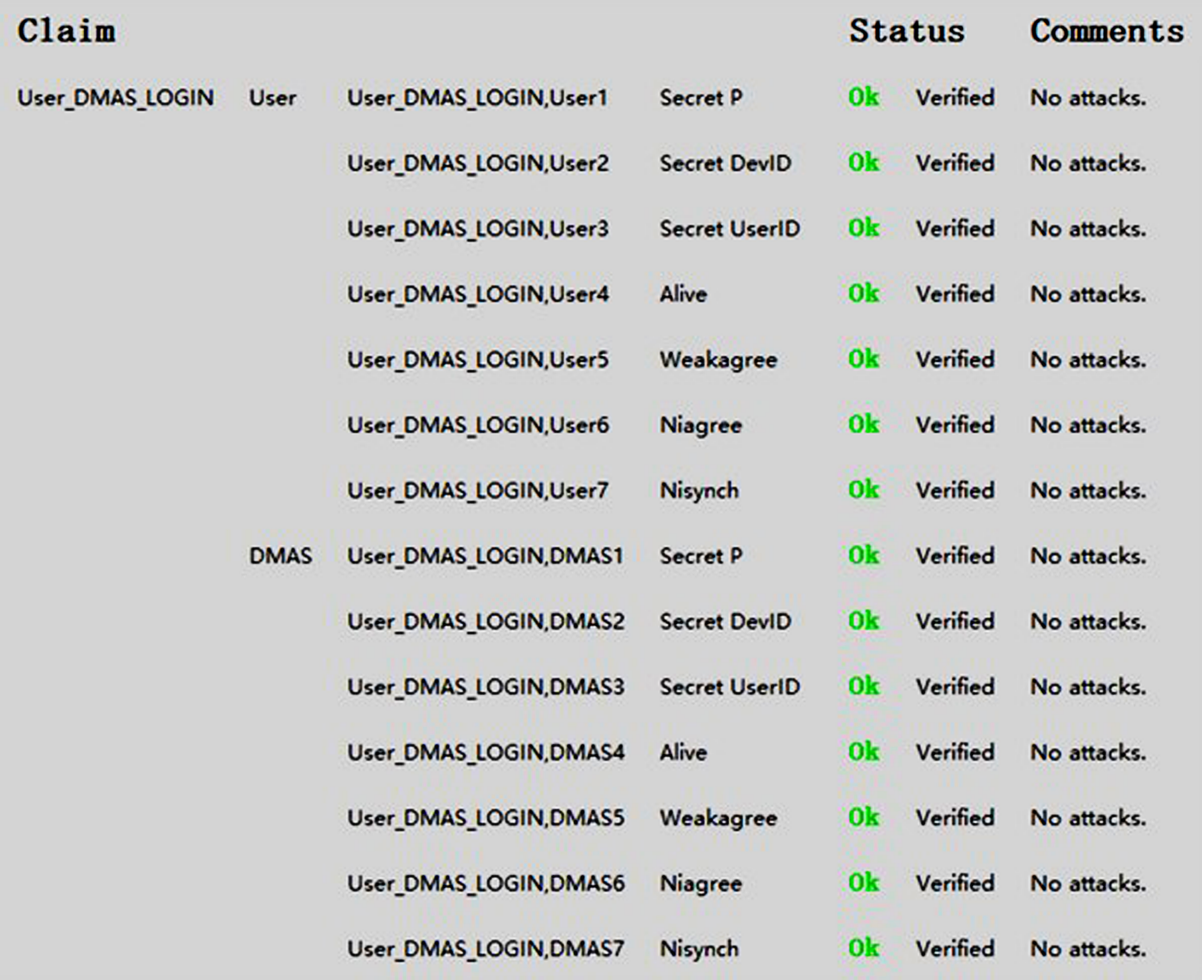
Formal analysis results from Scyther for the the login protocol. Between the user and the DMAS.

Then we use Scyther to analyze the login protocol between the user and the DMAS. The Scyther testing code is in Appendices B. Fig 6 illustrates the results obtained from Scyther’s analysis, and our scheme gets a good result.

**Fig 7:**
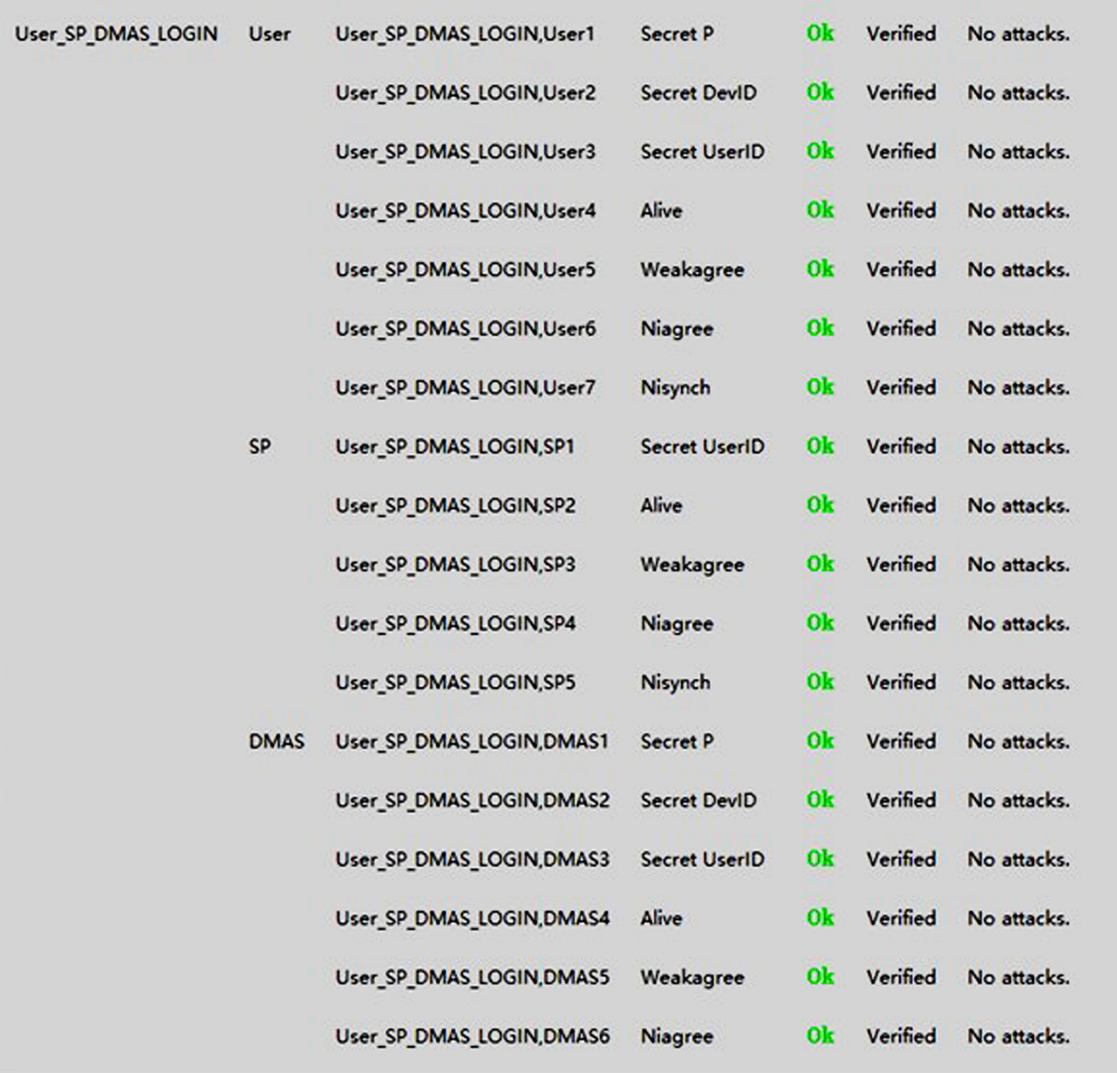
Formal analysis results from Scyther for the the login protocol. among the user, SP and DMAS.

Finally, we use Scyther to analyze the login protocol among the user, SP and DMAS whose UML sequence diagram is illustrated in Fig 4. The Scyther testing code is in Appendices C. Fig 7 illustrates the results obtained from Scyther’s analysis.

As can be seen, Scyther has been unable to determine any weaknesses or feasible attacks against our proposed scheme. We repeated the Scyther verification ten times, using both the manually defined claims and Scyther’s automatically generated claims, and the results remained the same.

Meanwhile, we also verified the security of our scheme from the point of view of cryptography information entropy.

The security of the password depends on the information entropy. The higher the information entropy, the higher the randomness, and the higher the security of the password. Suppose we set the number of pictures to *n*, the number of times the user needs to be challenged is *t*, the number of correct options in i round is *k*_*i*_, so the information entropy is

$$H=\log(\prod_{i=0}^{t}C_{n}^{k_{i}})$$ (1)

From the user experience point of view, the number of options should not be too many, 9–15 is appropriate. For security considerations, the correct number of options per round is at least 3, so you can get the minimum *H*_min_, and by probability, we can get the maximum *H*_max_ when $k_{i}=\left\lfloor \frac{n}{2}\right\rfloor$. The formulas are as follows:

$$H_{\min}=\log({\left(C_{n}^{3}\right)}^{t})$$ (2)

$$H_{\max}=\log({\left(C_{n}^{\left\lfloor \frac{n}{2}\right\rfloor }\right)}^{t})$$ (3)

Since the optional range of Arabic numerals is 0 to 9, the information entropy *H*_*num*_ of each Arabic numeral is 1. And the known keyboard can knock out 98 different characters, so the information entropy of each text password *H*_*char*_ is 2, and through calculation, we can draw the outcome for reaching the same security, the length each Arabic password and text password needs. Table 3 shows the outcome for reaching the same security with Arabic passwords and text passwords, the specification our scheme needs.

**Table 3:** The outcome for reaching the same security with Arabic passwords and text passwords, the specification our scheme needs. (n: the number of pictures, t: the number of times the User needs to be challenged).

n	t	H	Equivalent length of Arabic password	Equivalent length of text password
8	3	5.24~5.53	5	2
8	5	8.74~9.23	8~9	4
12	3	7.03~8.90	7~9	3
12	5	11.71~14.83	11~14	5~7
14	3	7.68~10.61	7~10	3~5
14	5	12.81~17.68	12~17	6~8
16	3	8.24~12.33	8~12	4~6
16	5	13.74~20.55	13~20	6~10

**Fig 8:**
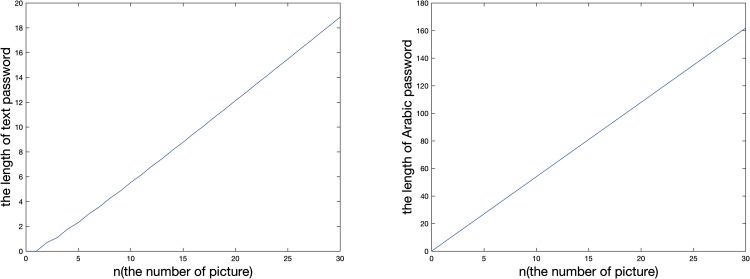
The intensity enhancement trend curve of text passwords and our scheme in mathematical statistics.

After that, we validate the intensity enhancement trend curve of text passwords and our scheme in mathematical statistics, as illustrated in Fig 8:

As can be seen from Fig 8, the intensity enhancement trend of Arabic passwords, text passwords and our scheme increases with the increase of the order of magnitude, and the growth slope of our scheme is greater than the growth slope of the Arabic passwords and text passwords.

Because the Arabic number password's information entropy is very weak, it can be cracked very quickly through a brute force password attack. As for text passwords, there are many possibilities. If it is pure uppercase and lowercase letters, its security is also weak. If it is a combination of numbers and letters, the security is more general, because text passwords are logical reasoning. For example, if someone's name is Jack, his password is more likely to be like Jack, or Jack plus a string of numbers (like a birthday). In our scheme, because of the User's own uploaded digital memory, there is no password to crack, because the brute force password attack requires a dictionary, and our scheme cannot create a crack dictionary because of the digital memory difference among users. Therefore, it is somewhat difficult to guess the answer through logical reasoning.

Furthermore, our scheme uses a large number of cryptographic protocols to ensure that attackers cannot enter the authentication page (such as DevID, timestamp and digital signature), so for attackers, obtaining the challenge and then cracking it is more difficult. Relative to the existing text password which anyone can access and attempt to crack, our security threshold is higher. Table 4 illustrates the password strength of Arabic number passwords, text passwords and our scheme.

**Table 4:** The strength of Arabic number passwords, text passwords and our scheme.

	High	Medium	Weak
Arabic number password			√
Text password		√	
Our scheme	√		

## Implementation and performance analysis

### Implementation

**Fig 9:**
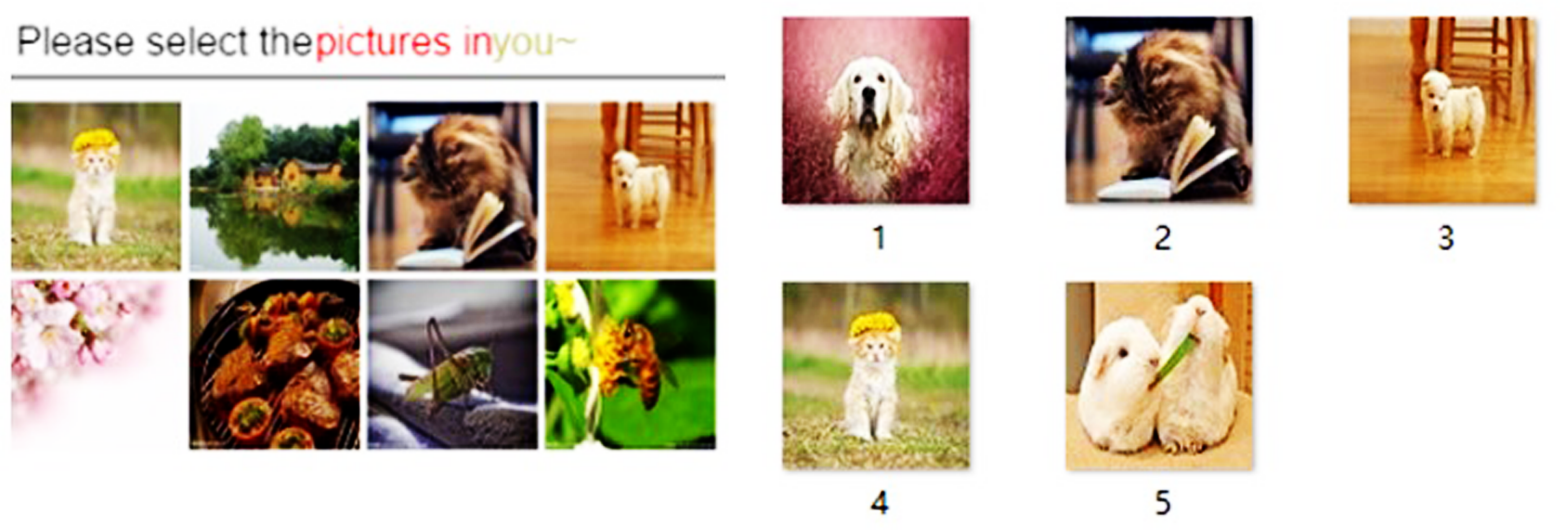
Example challenge of normal strictness degree and the pictures in picture group “you~”.

Our scheme is extensively applicable to all kinds of SPs with different strictness degree and anonymous degree requirements. For example, if the user wants to log in to an SP with a normal authentication strictness degree requirement, the DMAS is likely to give the user a challenge such as in Fig 9. The DMAS chooses a digital memory object group named “you~” from the user’s digital memories database, which is a proper subset of this group with random quantities displayed in a grid of eight digital memory objects. The name “you~” is a code word and only the user knows the meaning of the code word.

So in this example, the user uses the code word “you~” to refer to some pets pictures. Because of the random quantities, true answer digital memory objects are used and the distractors are also from the user’s digital memories, it’s difficult to guess the true answer.

**Fig 10:**
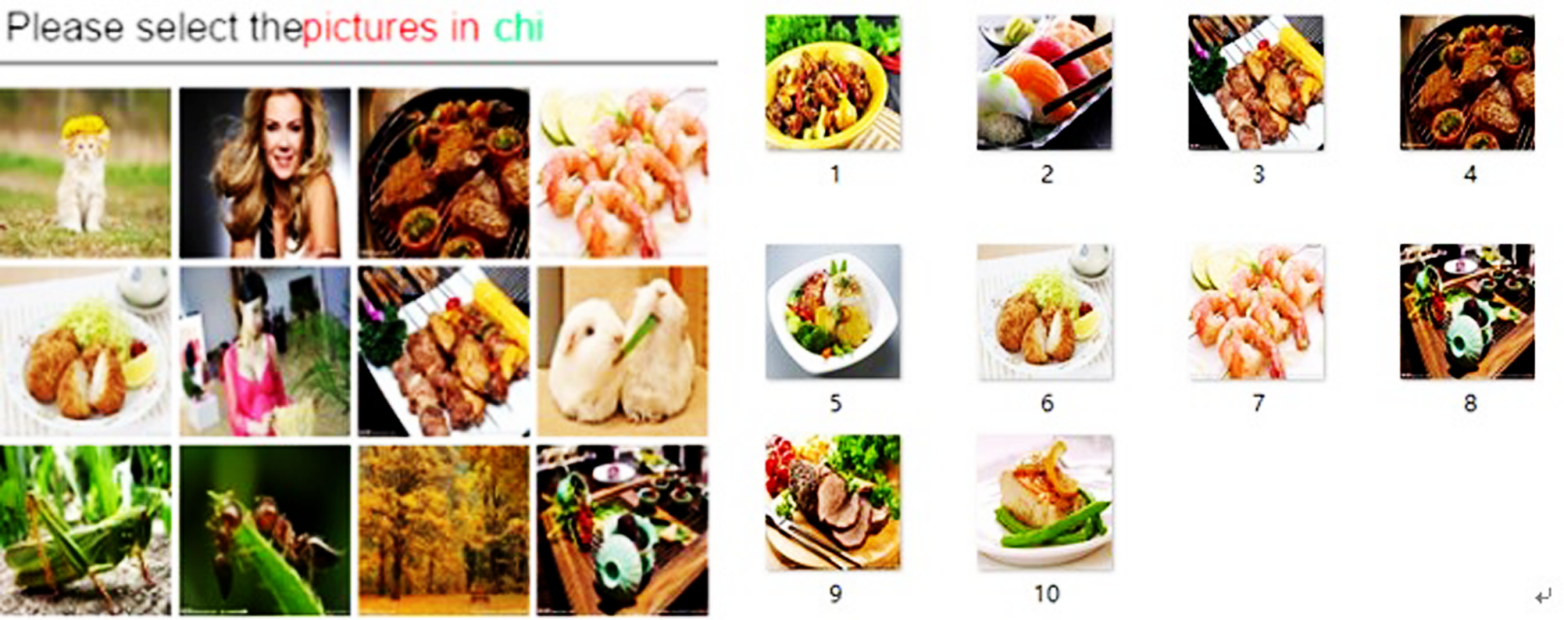
Example challenge of higher strictness degree and the pictures in picture group “chi”.

Another potential application area is for the SPs with higher authentication strictness degree requirements, such as online banking. The DMAS is likely to give the user a challenge such as in Fig 10. The DMAS chooses a digital memory objects group named “chi” from the User’s digital memories database, which is a proper subset of this group with random quantities displayed in a grid of twelve pictures. The name “chi” is also a code word and only the user knows the meaning of the code word.

So in this example, the user uses the code word “chi” to refer to some food digital memory object. In this way, guessing and shoulder surfing attacks will be more difficult.

### Performance analysis

#### The user registers with the DMAS

We test the registration phase with an Intel(R) Pentium(R) 4 [28] device as the user’s device, and the DMAS is a server equipped with Intel(R) Xeon(R) l5520[29]. We upload 10 digital memory objects as two groups, the outcome is as follows Table 5:

**Table 5:** Performance analysis of phase 2: The user registers with the DMAS (h-hash function; AE-Asymm Encrypt; SE-Symm Encrypt, AD- Asymm Decrypt, SD: Symm Decrypt).

Step	Time Used (User)	User	Time Used (DMAS)	DMAS	Total
1	0.5s	1AE	1s	1AD	1AE 1AD
2	2s	10SE 13AE 10h	1s	1AD	10SE 13AE 10h 1AD
3	3s	1AD	0.4s	1AE 1h	1AE 1AD 1h
Total	5.5s	10SE 14AE 10h 1AD	2.4s	2AD 1h 1AE	10SE 15AE 11h 3AD

#### The user logs in to the SP

In this phase, we use the same devices as in the registration phase for the user and the DMAS, then we use a second server equipped with Intel(R) Xeon(R) l5520[29] as the SP. The challenge includes one group and 5 digital memory objects used in the authentication step. The outcome is as follows Table 6:

**Table 6:** Performance analysis of phase 3: The user logs in to the SP (h-hash function; AE-Asymm Encrypt; SE-Symm Encrypt, AD- Asymm Decrypt, SD: Symm Decrypt).

Step	Time Used (User)	User	Time Used (DMAS)	DMAS	Time Used (SP)	SP	Total
1			1s	1AD	0.3s	1AE	1AE 1AD
2	0.5s	1AE	1s	1AD			1AE 1AD
3	3.4s	2AD 5SD	0.4s	1AE 1h			1AE 1h 2AD 5SD
4	0.5s	1AE	1s	1AD			1AE 1AD
5	3s	1AD	0.8s	2h 2AE	1s	1AD	2AE 2AD 2h
Total	7.4s	2AE 3AD 5SD	4.2s	3AD 3h 3AE	1.3s	1AE 1AD	6AE 7AD 3h 5SD

The tables show that the two phases cost little time, little enough to be acceptable in the authentication stage. Also, the CPUs used in our devices in the tests are now outdated, so we believe our scheme can perform much better with up to date devices.

## Conclusion and future directions

In this paper, we have presented a digital memories based user authentication scheme with privacy preservation. In our scheme, we adopt homomorphic encryption and public key encryption to achieve leak-proof user privacy, untrackable user behavior, and to provide multi-level security in Internet & IoT environments. Also, we prove the reliability and security of our scheme compared to other schemes and present performance analysis and promising evaluation results.

However, there are still some details that can be further optimized, such as if the user forgets his/her digital passwords or wants to edit the picture groups and upload new digital memories, the authentications used nowadays are email authentication or SMS authentication which demand the user receive an email or a SMS and inputs the verification code to verify if the user is legal; these methods will not be appropriate in the future.

In our future work, we are hoping to expand our initial implementation into a fully working prototype and to optimize the details. We also aim to continue further study into digital memories based user authentication and come up with more constructive ideas.

We have uploaded the scyther protocol analysis files, the scyther analyzer, and all the figures that appear in our manuscript as our support file, and anyone can use it without restriction.

### Appendices

**A**: **code of Scyther for the the registration protocol between User and DMAS**

hashfunction H1;

usertype Timestamp;

protocol User-DMAS-REG(User,DMAS)

{

        role User

        {

                const DevID: Nonce;

                const UserID: Nonce;

                const P: Nonce;

                const R-I: Nonce;

                fresh Ts: Timestamp;

                send_1(User,DMAS,{DevID,UserID,User}pk(DMAS));

                send_2(User,DMAS,{{P}k(User,DMAS),R-I,DevID,UserID,User,Ts}pk(DMAS));

                recv_3(DMAS,User,{H1(UserID,DevID,R-I),Ts,DMAS}sk(DMAS));

                claim(User,Secret,P);

                claim(User,Secret,R-I);

                claim(User,Secret,DevID);

                claim(User,Secret,UserID);

                claim(User,Alive);

                claim(User,Weakagree);

                claim(User,Niagree);

                claim(User,Nisynch);

        };

        role DMAS

        {

                var DevID: Nonce;

                var UserID: Nonce;

                var R-I: Nonce;

                var P: Nonce;

                fresh Ts: Timestamp;

                recv_1(User,DMAS,{DevID,UserID,User}pk(DMAS));

                recv_2(User,DMAS,{{P}k(User,DMAS),R-I,DevID,UserID,User,Ts}pk(DMAS));

                send_3(DMAS,User,{H1(UserID,DevID,R-I),Ts,DMAS}sk(DMAS));

                claim(DMAS,Secret,P);

                claim(DMAS,Secret,R-I);

                claim(DMAS,Secret,DevID);

                claim(DMAS,Secret,UserID);

                claim(DMAS,Alive);

                claim(DMAS,Weakagree);

                claim(DMAS,Niagree);

                claim(DMAS,Nisynch);

        };

};

**B**: **code of Scyther for the the login protocol between User and DMAS**

hashfunction H1;

usertype Timestamp;

protocol User-DMAS-LOGIN(User,DMAS)

{

        role User

        {

                const DevID: Nonce;

                const UserID: Nonce;

                const Ans: Nonce;

                fresh Ts: Timestamp;

                var Pass-flag: Nonce;

                var P: Nonce;

                send_1(User,DMAS,{UserID,DevID,User,Ts}pk(DMAS));

                recv_2(DMAS,User,{Ts,DMAS,H1(UserID),{P}k(User,DMAS)}sk(DMAS));

                send_3(User,DMAS,{DevID,UserID,User,Ans,Ts}pk(DMAS));

                recv_4(DMAS,User,{H1(UserID),Pass-flag,Ts}sk(DMAS));

                claim(User,Secret,P);

                claim(User,Secret,DevID);

                claim(User,Secret,UserID);

                claim(User,Alive);

                claim(User,Weakagree);

                claim(User,Niagree);

                claim(User,Nisynch);

        };

        role DMAS

        {

                const P: Nonce;

                const Pass-flag: Nonce;

                fresh Ts: Timestamp;

                var DevID: Nonce;

                var UserID: Nonce;

                var Ans: Nonce;

                recv_1(User,DMAS,{UserID,DevID,User,Ts}pk(DMAS));

                send_2(DMAS,User,{Ts,DMAS,H1(UserID),{P}k(User,DMAS)}sk(DMAS));

                recv_3(User,DMAS,{DevID,UserID,User,Ans,Ts}pk(DMAS));

                send_4(DMAS,User,{H1(UserID),Pass-flag,Ts}sk(DMAS));

                claim(DMAS,Secret,P);

                claim(DMAS,Secret,DevID);

                claim(DMAS,Secret,UserID);

                claim(DMAS,Alive);

                claim(DMAS,Weakagree);

                claim(DMAS,Niagree);

                claim(DMAS,Nisynch);

        };

};

**C**: **code of Scyther for the the login protocol among User, SP and DMAS**

hashfunction H1;

usertype Timestamp;

protocol User-SP-DMAS-LOGIN(User,SP,DMAS)

{

        role User

        {

                const DevID: Nonce;

                const UserID: Nonce;

                const Ans: Nonce;

                fresh Ts: Timestamp;

                var Pass-flag: Nonce;

                var P: Nonce;

                send_2(User,DMAS,{UserID,DevID,User,Ts}pk(DMAS));

                recv_3(DMAS,User,{Ts,DMAS,H1(UserID),{P}k(User,DMAS)}sk(DMAS));

                send_4(User,DMAS,{DevID,UserID,User,Ans,Ts}pk(DMAS));

                recv_5(DMAS,User,{H1(UserID),Pass-flag,Ts}sk(DMAS));

                claim(User,Secret,P);

                claim(User,Secret,DevID);

                claim(User,Secret,UserID);

                claim(User,Alive);

                claim(User,Weakagree);

                claim(User,Niagree);

                claim(User,Nisynch);

        };

        role SP

        {

                const PartnerID: Nonce;

                fresh Ts: Timestamp;

                var UserID: Nonce;

                send_1(SP,DMAS,{SP,PartnerID,Ts}pk(DMAS));

                recv_6(DMAS,SP,{H1(PartnerID),UserID,Ts}sk(DMAS));

                claim(SP,Secret,UserID);

                claim(SP,Alive);

                claim(SP,Weakagree);

                claim(SP,Niagree);

                claim(SP,Nisynch);

        };

        role DMAS

        {

                const P: Nonce;

                const Pass-flag: Nonce;

                fresh Ts: Timestamp;

                var DevID: Nonce;

                var UserID: Nonce;

                var Ans: Nonce;

                var PartnerID: Nonce;

                recv_1(SP,DMAS,{SP,PartnerID,Ts}pk(DMAS));

                recv_2(User,DMAS,{UserID,DevID,User,Ts}pk(DMAS));

                send_3(DMAS,User,{Ts,DMAS,H1(UserID),{P}k(User,DMAS)}sk(DMAS));

                recv_4(User,DMAS,{DevID,UserID,User,Ans,Ts}pk(DMAS));

                send_5(DMAS,User,{H1(UserID),Pass-flag,Ts}sk(DMAS));

                send_6(DMAS,SP,{H1(PartnerID),UserID,Ts}sk(DMAS));

                claim(DMAS,Secret,P);

                claim(DMAS,Secret,DevID);

                claim(DMAS,Secret,UserID);

                claim(DMAS,Alive);

                claim(DMAS,Weakagree);

                claim(DMAS,Niagree);

                claim(DMAS,Nisynch);

        };

};

## Supporting information

S1 File Scyther protocol analysis file: User-DMAS-LOGIN.spdl. (SPDL)

S2 File Scyther protocol analysis file: User-DMAS-reg.spdl. (SPDL)

S3 File Scyther protocol analysis file: User-DMAS-SP LOGIN.spdl. (SPDL)

S4 File The scyther analyzer: scyther-w32-v1.1.3. (ZIP)
