# Supplementary figures and images for: A digital memories based user authentication scheme with privacy preservation
Source: PLoS One. 2017 Nov 30;12(11):e0186925. doi: 10.1371/journal.pone.0186925 (PMC5708710; doi:10.1371/journal.pone.0186925)

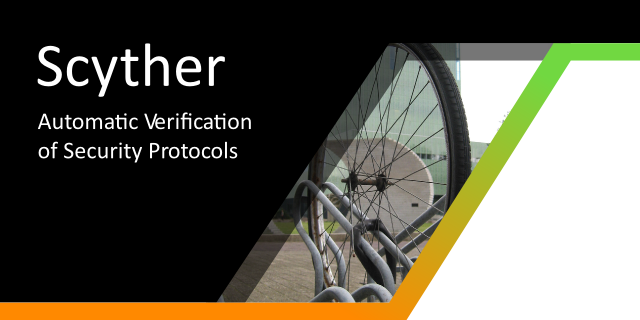

Supplement: S4 File — (ZIP) [file pone.0186925.s004.zip › S4_File/scyther-w32-v1.1.3/Images/scyther-splash.png]
