# Supplementary material for: A digital memories based user authentication scheme with privacy preservation
Source: PLoS One. 2017 Nov 30;12(11):e0186925. doi: 10.1371/journal.pone.0186925 (PMC5708710; doi:10.1371/journal.pone.0186925)
Supplement: S4 File — (ZIP) [file pone.0186925.s004.zip › S4_File/scyther-w32-v1.1.3/scyther-manual.pdf]

Cas Cremers

Scyther

User Manual

Draft February 18, 2014

Contents

1 Introduction **5**

2 Background **7**

3 Installation **9**

4 Quick start tutorial **11**

5 Input Language **15**
5.1 A minimal input file . . . 15
5.2 Terms . . . 15
5.2.1 Atomic terms . . . 15
5.2.2 Pairing . . . 16
5.2.3 Symmetric keys . . . 16
5.2.4 Asymmetric keys . . . 17
5.2.5 Hash functions . . . 17
5.2.6 Predefined types . . . 17
5.2.7 Usertypes . . . 17
5.3 Events . . . 18
5.3.1 Receive and send events . . . 18
5.3.2 Claim events and Security properties . . . 18
5.3.3 Internal computation/pattern match events . . . 19
5.4 Role definitions . . . 20
5.5 Protocol definitions . . . 20
5.6 Global declarations . . . 21
5.7 Miscellaneous . . . 21
5.7.1 Macro . . . 21
5.7.2 Include . . . 22
5.7.3 one-role-per-agent . . . 23
5.8 Language BNF . . . 23
5.8.1 Input file . . . 23
5.8.2 Protocols . . . 23
5.8.3 Roles . . . 23
5.8.4 Events . . . 24
5.8.5 Declarations . . . 24
5.8.6 Terms . . . 24

6 Modeling security protocols **25**
6.1 Introduction . . . 25
6.2 Example: Needham-Schroeder Public Key . . . 25

7 Specifying security properties **29**
7.1 Specifying secrecy . . . 29
7.2 Specifying authentication properties . . . 29
7.2.1 Aliveness . . . 29
7.2.2 Non-injective synchronisation . . . 29
7.2.3 Non-injective agreement . . . 29
7.2.4 Agreement over data . . . 29

8 Using the Scyther tool GUI **31**
8.1 Results . . . 31
8.2 Bounding the statespace . . . 33
8.3 Attack graphs . . . 33
8.3.1 Runs . . . 33
8.3.2 Communication events . . . 35
8.3.3 Claims . . . 36

9 Using the Scyther command-line tools **37**

10 Advanced topics **39**
10.1 Modeling more than one asymmetric key pair . . . 39
10.2 Approximating equational theories . . . 39
10.3 Modeling time-stamps and global counters . . . 41
10.3.1 Modeling global counters . . . 41
10.3.2 Modeling time-stamps using nonces . . . 41
10.3.3 Modeling time-stamps using variables . . . 42
10.4 Multi-protocol attacks . . . 42

11 Further reading **45**

A Full specification for Needham-Schroeder public key **49**

Chapter 1

Introduction

> **Note:** This is a draft of the new version of the Scyther manual. The manual may therefore be incomplete at points.
> Any feedback is welcome and can be sent to Cas Cremers by e-mail: `cas.cremers@cs.ox.ac.uk`.

This is the user manual for the Scyther security protocol verification tool.

The purpose of this manual is to explain the details of the Scyther input language, explain how to model basic protocols, and how to effectively use the Scyther tool. This manual does not detail the protocol execution model nor the adversary model used by the tool. It is therefore highly recommended to read the accompanying book [1]. The book includes a detailed description of Scyther's underlying protocol model, security property specifications, and the algorithm.

We proceed in the following way. Some background is given in Chapter 2. Chapter 3 explains how to install the Scyther tool on various platforms. In Chapter 4 we give a brief tutorial using simple examples to show the basics of the tool. Then we discuss things in more detail as we introduce the input language of the tool in Chapter 5. Modeling of basic protocols is described in Chapter 6, and Chapter 7 describes how to specify security properties. The usage of the GUI version of tool is then explained in more detail in Section 8. The underlying command-line tool is described in Section 9. Advanced topics are discussed in Section 10.

Online information

More help can be found online on the Scyther website:

`http://users.ox.ac.uk/~coml0529/scyther/index.html`

Users are advised to subscribe to the Scyther mailing list, whose details can also be found on the Scyther website.

Chapter 2

Background

Scyther is a tool for the formal analysis of security protocols under the *perfect cryptography assumption*, in which it is assumed that all cryptographic functions are perfect: the adversary learns nothing from an encrypted message unless he knows the decryption key. The tool can be used to find problems that arise from the way the protocol is constructed. This problem is undecidable in general, but in practice many protocols can be either proven correct or attacks can be found.

The full protocol model, its assumptions, the basic security properties, and the algorithm are described in [1]. This manual serves as a companion to the book. Thus, in this manual we assume the reader is familiar with the formal modeling of security protocols and their properties.

Chapter 3

Installation

Scyther can be downloaded from the following website:
`http://users.ox.ac.uk/~coml0529/scyther/`

Installation instruction are included in the downloadable Scyther archives. Scyther is available for the Windows, Linux and Mac OS platforms.

Chapter 4

Quick start tutorial

Scyther takes as input a security protocol description that includes a specification of intended security properties, referred to as security claims, and evaluates these.

Start Scyther by executing the `scyther-gui.py` program in the Scyther directory. The program will launch two windows: the main window, in which files are edited, and the `about` window, which shows some information about the tool.

As an introductory example, we will verify the Needham-Schroeder protocol, and investigate an attack on it.

Go to the file→open dialog, and open the file `ns3.spdl` in the Scyther directory. Your main window should look like the one in Figure 4.

By convention, protocol description files have the extension `.spdl` (Security Protocol Description Language), but it can have any name. The file used in this example is shown in Appendix A.

Run the verification tool by selecting verify→verify_claims in the menu. A new window will appear during the verification process. Once verification is completed, the window will be replaced by the result window, as shown in Figure 4.

The result window shows a summary of the claims in the protocol, and the verification results. Here one can find whether the protocol is correct, or false. In the next section there will be a full explanation of the possible outcomes of the verification process. Most importantly, if a protocol claim is incorrect, then there exists at least one attack on the protocol. A button is shown next to the claim: press this button to view the attacks on the claim, as in Figure 4.

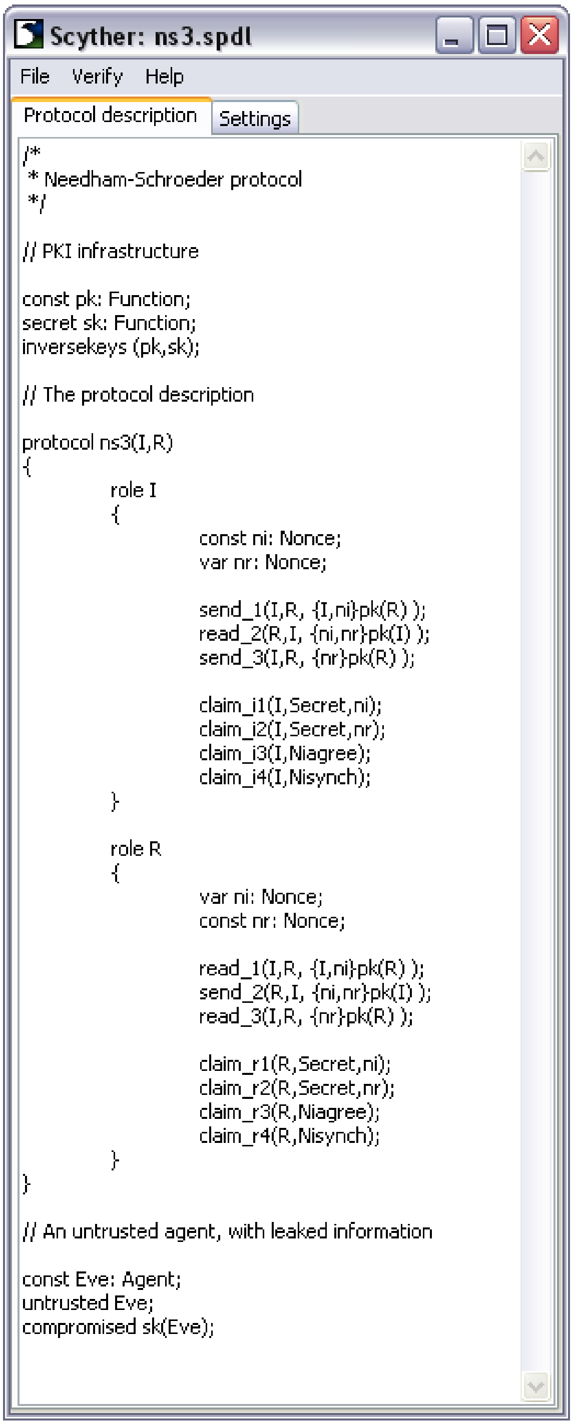

Figure 4.1: Scyther main window with the file ns3.spdl opened

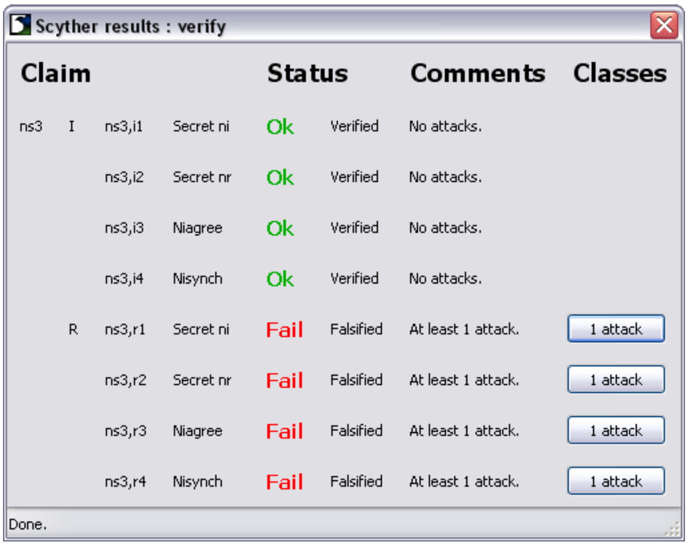

Figure 4.2: Scyther result window

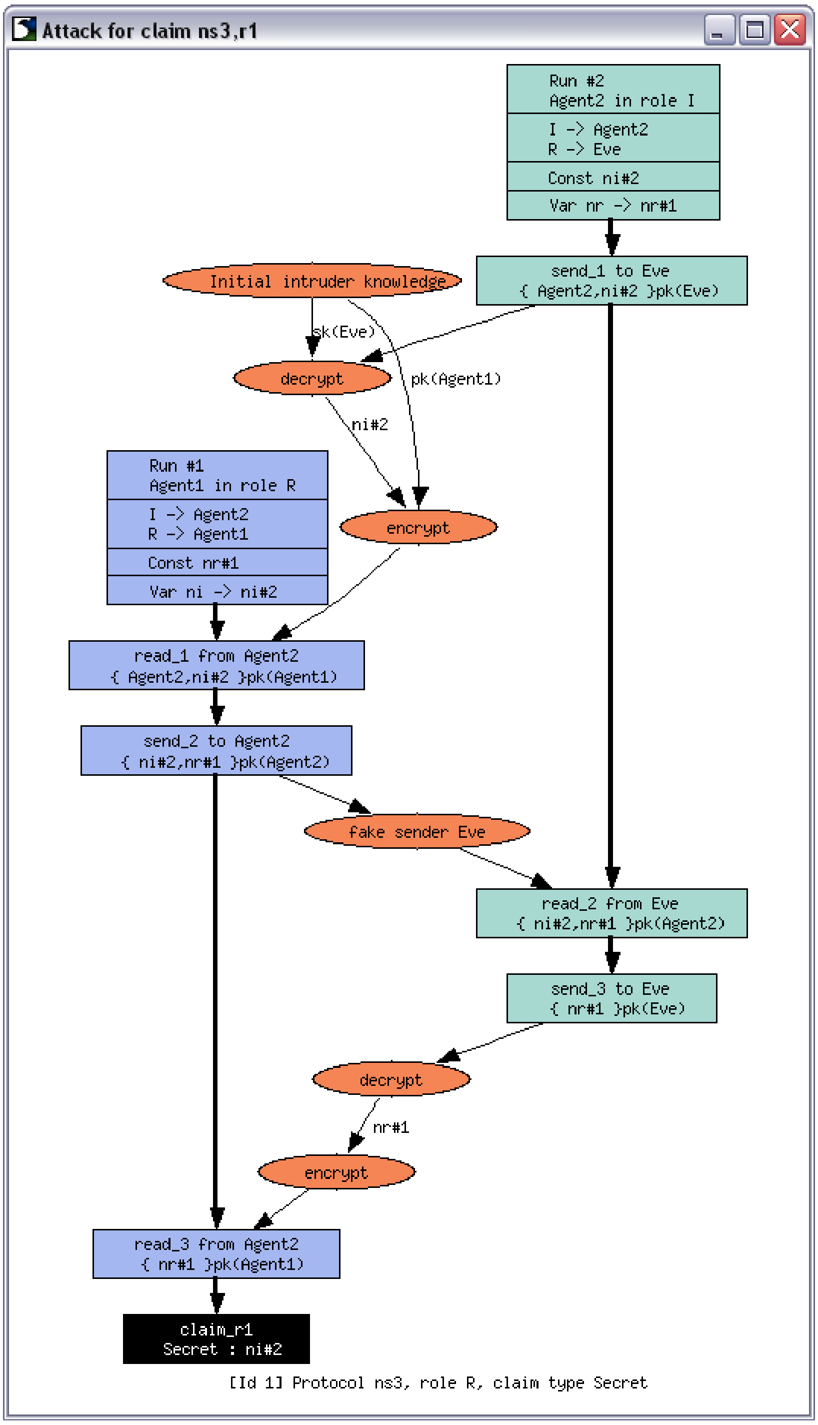

Figure 4.3: Scyther attack window

Chapter 5

Input Language

Scyther's input language is loosely based on a C/Java-like syntax. The main purpose of the language is to describe protocols, which are defined by a set of roles. Roles, in turn, are defined by a sequence of events, most of which are events that denote the sending or receiving of terms. We describe these elements in the following sections.

Comments can start with `//` or `#` (for single-line comments) or be enclosed by `/*` and `*/` (for multi-line comments). Note that multi-line comments cannot be nested.

Any whitespace between elements is ignored. It is therefore possible to use whitespace (spaces, tabs, newlines) to improve readability.

A basic identifier consists of a string of characters from the set of alphanumeric characters as well as the symbols `^` and `-`.

The language is case-sensitive, thus `NS3` is not the same identifier as `ns3`.

5.1 A minimal input file

The core elements in a Scyther input file are protocol definitions. A minimal example is the following:

```
protocol ExampleProtocol(I,R) {
  role I { };
  role R { };
};
```

In the above, we have defined a protocol called "ExampleProtocol" that has two roles, "I" and "R" by listing them between brackets after the protocol name. Note that we haven't defined the behaviour of these roles yet: such behaviours are defined within the curly brackets after the corresponding `role I` and `role R` commands.

5.2 Terms

At the most basic level, Scyther manipulates terms.

5.2.1 Atomic terms

An atomic term can be any identifier, which is usually a string of alphanumeric characters.

Atomic terms can be combined into more complex terms by operators such as pairing and encryption.

Constants

Freshly generated values

Many security protocols rely on generating random values. They can be specified by declaring them inside a role definition using the `fresh` declaration. For example, to generate a random value `Na` of type `Nonce`, we specify:

```
role X(...) {
  fresh Na: Nonce;

  send_1(X,Y,Na);
}
```

Variables

Agents can use variables to store received terms. For example, to receive a nonce into a variable with name `Na`, we specify:

```
role Y(...) {
  var Na: Nonce;

  recv_1(X,Y,Na);
}
```

Local declarations, for both freshly generated values as well as variables such as `Na`, are local to the role. Thus, one can specify a *freshly generated nonce* `Na` in one role and a variable `Na` in another role without any conflicts. Variables are rigid: after the first receive event in which they occur has been executed, they are assigned a value. This value cannot be changed afterwards.

Variables must occur first in receive events: it is not allowed to use uninitialized variables in send events.

5.2.2 Pairing

Any two terms can combined into a term pair: we write `(x,y)` for the pair of terms `x` and `y`. It is also allowed to write n-tuples as `(x,y,z)`, which is interpreted by Scyther as `((x,y),z)`.

5.2.3 Symmetric keys

Any term can act as a key for symmetrical encryption.

The encryption of `ni` with a term `kir` is written as:

```
{ ni }kir
```

Unless `kir` is explicitly defined as being part of an asymmetric key pair (explained below), this is interpreted as symmetric encryption.

A symmetric-key infrastructure is predefined: `k(X,Y)` denotes the long-term symmetric key shared between X and Y.

5.2.4 Asymmetric keys

A public-key infrastructure (PKI) is predefined: `sk(X)` denotes the long-term private key of X, and `pk(X)` denotes the corresponding public key.

As an example, consider the following term. It represents the encryption of some term `ni` by the term `pk(I)`. Under normal conventions, this means that the nonce of the initiator (`ni`) is encrypted with the public key of the initiator.

```
{ ni }pk(I)
```

This term can only be decrypted by an agent who knows the secret key `sk(I)`.

Section 10.1 describes how to model more than one key pair per agent.

5.2.5 Hash functions

Hash functions are essentially encryptions with a function, of which the inverse is not known by anybody.

They can be used by a global declaration of an identifier to be a hashfunction, e. g.:

```
hashfunction H1;
```

As all agents and protocols should have access to such a function, the declaration of hashfunction is usually global, i. e., defined outside of any protocol definition.

Once declared, they can be used in protocol messages, e. g.:

```
H1(ni)
```

5.2.6 Predefined types

`Agent` Type used for agents.

`Function` A special type that defines a function term that can take a list of terms as parameter. By default, it behaves like a hash function: given the term `h(x)` where `h` is of type `Function`, it is impossible to derive `x`.

`Nonce` A standard type that is often used and therefore defined inside the tool.

`Ticket` A variable of type `Ticket` can be substituted by any term.

5.2.7 Usertypes

It is possible to define a new type. This can be done using the `usertype` command:

```
usertype MyAtomicMessage;

protocol X(I,R) {
  role I {
    var y: MyAtomicMessage;

    recv_1(I,R, y );
```

The effect of such a declaration is that variables of the new type can only be instantiated with messages `m` of that type, i. e., that have been declared by the global declaration `const m: MyAtomicMessage` or the freshly generated `fresh m: MyAtomicMessage` within a role.

In general, the tool can perform better if more is known about which messages might unify or not. By defining a usertype, the modeler can inform the tool that a variable can only be instantiated with terms of that type, and not with, e. g., terms of type Nonce. Conceptually, one can always write `Ticket` (which corresponds to all possible messages) for each variable type, but then one may find false attacks (in case the implementation in fact does check the type of a message) and the tool will be less likely to verify the property (for an unbounded number of runs).

5.3 Events

5.3.1 Receive and send events

The `recv` and `send` events mark receiving and sending a message, respectively.

Note that in some protocol description files one may find the `read` keyword: this is obsolete syntax and can safely be substituted by `recv`.

In most cases, each `send` event will have a corresponding `recv` event. We specify this correspondence by giving such events the same label, denoted by a subscript.

For some protocols we may want to model sending or receiving to the adversary directly, in which case we have no corresponding event. If a `send` or `recv` event has no corresponding event, Scyther will output a warning. To surpress this warning, the label can be prefixed by a bang `!`, e. g.:

```
send_!1(I,I, LeakToAdversary );
```

5.3.2 Claim events and Security properties

Claim events are used in role specifications to model intended security properties. For example, the following claim event models that `Ni` is meant to be secret.

```
claim(I, Secret, Ni);
```

There are several predefined claim types.

`Secret` This claim requires a parameter term. Secrecy of this term is claimed as defined in [1].

`SKR` The verification condition for this claim is equivalent to the `Secret` claim.

The purpose of this claim is to additionally mark the parameter term as a session-key. The consequence is that using the *session-key reveal* adversary rule will now reveal the parameter term.

If the *session-key reveal* rule is not enabled, this claim is identical to the `Secret` claim.

`Alive` Aliveness (of all roles) as defined in [4].

`Weakagree` Weak agreement (of all roles) as defined in [4].

`Commit, Running` Non-injective agreement with a role on a set of data items [4] can be defined by inserting the appropriate signal claims. In this context, `Commit` marks the effective claim, whose correctness requires the existence of a corresponding `Running` signal in the trace.

These claims are used to model agreement over data, which is explained in Section 7.2.4.

`Nisynch` Non-injective synchronisation as defined in [1].

`Niagree` Non-injective agreement on messages as defined in [1].

`Reachable` When this claim is verified, Scyther will check whether this claim can be reached at all. It is true iff there exists a trace in which this claim occurs. This can be useful to check if there is no obvious error in the protocol specification, and is in fact inserted when the `--check` mode of Scyther is used.

`Empty` This claim will not be verified, but simply ignored. It is only useful when Scyther is used as a back-end for other verification means. For more on this, see Section 10.

5.3.3 Internal computation/pattern match events

We extend the basic set of events from [1] with two events that can be used to model internal computations.

Match event

New in version v1.1 and Compromise-0.8

The first new event is the `match` event, that is used to specify pattern matching, i. e.,

```
match(pt,m)
```

In operational terms, if there exists a well-typed substitution σ such that $\sigma pt = m$, then this event can be executed. Upon execution, the substitution is applied to the remaining events of the role.

This event can be used to model various constructions, such as equality tests, delayed decryption, checking commitments. They can also be used to model internal computations to simplify specifications, e. g.:

```
var X: Nonce;
var Y;

recv(R,I, X);
match(Y, hash(X,I,R) );
send(I,R, Y,{ Y }sk(I) );
```

In the above example, we could have replaced `Y` by `hash(X,I,R)` throughout the specification, but this version avoid replication.

Not match event

New in version v1.1 and Compromise-0.8

The second new event is the `not match` event, that is used to specify pattern matching, i. e.,

```
not match(pt,m)
```

The operational interpretation is the opposite of the previous event. If there is no substitution σ such that $\sigma pt = m$, then the event can be executed.

This event can be used to model, e. g., inequality constraints. For example, the execution model allows by default agents executing sessions with themselves. In some cases, we want to exclude such behaviour, because the protocol disallows it. For example,

```
role A {
  not match(A,B);
  send (A,B, m1);
}
```

models a role whose instances only send messages to other agents.

As a more advanced usage, `match` and `not match` can be used together in two roles with a common starting sequence of events to model *if ... then ... else* constructions.

5.4 Role definitions

Role definitions are sequences of events, i. e., declarations, send, receive, or claim events.

```
role Server {
  var x,y,z: Nonce;
  fresh n,m: Nonce;

  send_1(Server,Init, m,n );
  recv_2(Init,Server, x,y, { z }pk(Server) );
}
```

5.5 Protocol definitions

A protocol definition takes as a parameter a sequence of roles, which are then defined within its body.

```
protocol MyProt(Init,Resp,Server)
{
  role Init {
    ...
  }
  role Resp {
    ...
  }
  role Server {
    ...
  }
}
```

Helper protocols

It is possible to prepend an “@” symbol before a protocol name. This has no effect on the protocol model, nor on the outcome of the analysis. The “@” is only used when rendering output graphs: the intent is to mark the protocol as a “helper protocol”. Such protocols are often used to model

additional adversary capabilities, see Section 10 for examples. When rendering output graphs, Scyther collapses role instances of helper protocols into single nodes. This can make the graphs more readable.

Symmetric-role protocols

Some adversary-compromise rules, such as SKR and $LKR_{aftercorrect}$ depend on a partnering function. For protocols that are entirely symmetric in their roles and key computations (such as HMQV), this is not the appropriate partnering function. To use the correct partnering function, the protocol needs to be annotated as a *symmetric-role* protocol. This instructs Scyther to use the appropriate partnering function.

```
symmetric-role protocol MyProt(Init,Resp)
{
  role Init {
    ...
  }
  role Resp {
    ...
  }
}
```

5.6 Global declarations

In many applications global constants are used. These include, for example, string constants, labels, or protocol identifiers.

They are modeled and used in the following way:

```
usertype String;
const HelloWorld: String;

protocol hello(I,R)
{
  role I {
    send_1(I,R, HelloWorld);
  }
  role R {
    recv_1(I,R, HelloWorld);
  }
}
```

5.7 Miscellaneous

5.7.1 Macro

New in version v1.1 and Compromise-0.8

It is possible to define *macros*, i. e., abbreviations for particular term. The syntax used to define these abbreviations is the following:

```
  macro MyShortCut = LargeTerm;
```

For example, for a protocol that contains complex messages or repeating elements, macros can be used to simplify the protocol specification:

```
hashfunction h;

protocol macro-example-one(I,R) {
   role I {
      fresh nI: Nonce;
      macro m1 = h(I,ni);

      send_1(I,R, { m1 }pk(R) );
      claim(I, Secret, m1);
   }
   role R {
      var X: Ticket;

      recv_1(I,R, { X }pk(R) );
   }
}
```

Note that macros have *global scope*, and are handled at the *syntactical* level. This also allows for global abbreviations of protocol messages, e. g.:

```
hashfunction h;
macro m1 = { I,R, nI, h(nI,R) }pk(R);

protocol macro-example-two(I,R) {
   role I {
      fresh nI: Nonce;

      send_1(I,R, m1 );
   }
   role R {
      var nI: Nonce;

      recv_1(I,R, m1 );
   }
}
```

Note that in the above example, `nI` is a freshly generated nonce in the I role, and a variable in the R role. Because the macro definitions are unfolded syntactically, the same macro can be used to refer to both terms.

5.7.2 Include

It is possible to import other files in a protocol specification:

```
include "filename";
```

where *filename* denotes the name of the file that will be included at this point. Using this command, it is possible to share, e. g., a set of common definitions between files. Typically this will include definitions for the key structures, and (untrusted) agent names. Nested use of this command is possible.

5.7.3 one-role-per-agent

New in version v1.1 and Compromise-0.8

The operational semantics allow agents to perform any roles, and even multiple different roles in parallel. This modeling choice corresponds to the worst possible scenario, in which the adversary has the most options to exploit. However, in many concrete settings, agents perform only one role. For example, the set of servers may be disjoint from the set of clients, or the set of RFID tags may be disjoint from the set of readers. In such cases, we do not need to consider attacks that exploit that an agent can perform multiple roles. This can be modeled by the following statement:

```
option "--one-role-per-agent"; // disallow agents in multiple roles
```

This causes Scyther to ignore attacks in which agents perform multiple roles. Phrased differently, this corresponds to the situation in which each role is performed by a dedicated set of agents.

5.8 Language BNF

The full BNF grammar for the input language is given below. In the strict language definition, there are no claim terms such as `Niagree` and `Nisynch`, and neither are there any predefined type classes such as `Agent`. Instead, they are predefined constant terms in the Scyther tool itself.

5.8.1 Input file

An input file is simply a list of spdl constructions, which are global declarations or protocol descriptions.

⟨*spdlcomplete*⟩ ::= ⟨*spdl*⟩ { ';' ⟨*spdl*⟩ }

⟨*spdl*⟩ ::= ⟨*globaldeclaration*⟩
  | ⟨*protocol*⟩

5.8.2 Protocols

A protocol is simply a container for a set of roles. Because we use a role-based approach to describing roles, the protocol container in fact only affects the naming of the roles: a role "I" in a protocol "ns3" will internally be assigned the name "ns3.I". This is used to make role names globally unique.

⟨*protocol*⟩ ::= '`protocol`' ⟨*id*⟩ '`(`' ⟨*termlist*⟩ '`)`' '`{`' ⟨*roles*⟩ '`}`' ['`;`']

5.8.3 Roles

⟨*roles*⟩ ::= ⟨*role*⟩ [⟨*roles*⟩]
  | ⟨*declaration*⟩ [⟨*roles*⟩]

⟨*role*⟩ ::= ['`singular`'] '`role`' ⟨*id*⟩ '`{`' ⟨*roledef*⟩ '`}`' ['`;`']

⟨*roledef*⟩ ::= ⟨*event*⟩ [⟨*roledef*⟩]
  | ⟨*declaration*⟩ [⟨*roledef*⟩]

5.8.4 Events

⟨*event*⟩ ::= ‘`recv`’ ⟨*label*⟩ ‘`(`’ ⟨*from*⟩ ‘`,`’ ⟨*to*⟩ ‘`,`’ ⟨*termlist*⟩ ‘`)`’ ‘`;`’
  | ‘`send`’ ⟨*label*⟩ ‘`(`’ ⟨*from*⟩ ‘`,`’ ⟨*to*⟩ ‘`,`’ ⟨*termlist*⟩ ‘`)`’ ‘`;`’
  | ‘`claim`’ [⟨*label*⟩] ‘`(`’ ⟨*from*⟩ ‘`,`’ ⟨*claim*⟩ [‘`,`’ ⟨*termlist*⟩] ‘`)`’ ‘`;`’

⟨*label*⟩ ::= ‘`_`’ ⟨*term*⟩

⟨*from*⟩ ::= ⟨*id*⟩

⟨*to*⟩ ::= ⟨*id*⟩

⟨*claim*⟩ ::= ⟨*id*⟩

5.8.5 Declarations

⟨*globaldeclaration*⟩ ::= ⟨*declaration*⟩
  | ‘`untrusted`’ ⟨*termlist*⟩ ‘`;`’
  | ‘`usertype`’ ⟨*termlist*⟩ ‘`;`’

⟨*declaration*⟩ ::= [‘`secret`’] ‘`const`’ ⟨*termlist*⟩ [‘`:`’ ⟨*type*⟩] ‘`;`’
  | [‘`secret`’] ‘`fresh`’ ⟨*termlist*⟩ [‘`:`’ ⟨*typelist*⟩] ‘`;`’
  | [‘`secret`’] ‘`var`’ ⟨*termlist*⟩ [‘`:`’ ⟨*typelist*⟩] ‘`;`’
  | ‘`secret`’ ⟨*termlist*⟩ [⟨*type*⟩] ‘`;`’
  | ‘`inversekeys`’ ‘`(`’ ⟨*term*⟩ ‘`,`’ ⟨*term*⟩ ‘`)`’ ‘`;`’
  | ‘`compromised`’ ⟨*termlist*⟩ ‘`;`’

⟨*type*⟩ ::= ⟨*id*⟩

⟨*typelist*⟩ ::= ⟨*type*⟩ { ‘`,`’ ⟨*type*⟩ }

5.8.6 Terms

⟨*term*⟩ ::= ⟨*id*⟩
  | ‘`{`’ ⟨*termlist*⟩ ‘`}`’ ⟨*key*⟩
  | ‘`(`’ ⟨*termlist*⟩ ‘`)`’
  | ⟨*id*⟩ ‘`(`’ ⟨*termlist*⟩ ‘`)`’

⟨*key*⟩ ::= ⟨*term*⟩

⟨*termlist*⟩ ::= ⟨*term*⟩ { ‘`,`’ ⟨*term*⟩ }

Chapter 6

Modeling security protocols

6.1 Introduction

The correct modeling of a security protocol for analysis in the Scyther tool requires a basic understanding of the underlying symbolic model. This model is explained in detail in [1].

Roughly speaking, the symbolic analysis focuses on the following aspects:

- Logical message components and their intended function within the protocol (public versus secret, freshly generated in each run or constant)
- Message structure (pairing, encryption, signing, hashing)
- Message flow (order, involved agents)

Many other elements are abstracted away. For example, bit strings are abstracted into terms, bit strings that occur with negligble probability are abstracted away, and more complext control flow constructs such as loops are often unfolded for a (low) finite number of times.

6.2 Example: Needham-Schroeder Public Key

As an example, we show how to model a simple protocol.

Figure 6.1 depicts the Needham-Schroeder Public Key protocol. For simplicity, we have only displayed the claim by each role that the initiator nonce ni is secret.

We start off the protocol description by adding a multi-line comment that describes the protocol and other interesting details. Multi-line comments start with `/*` and end with `*/`.

```
/*
 * Needham-Schroeder protocol
 */
```

The protocol uses the default public/private key infrastructure: an agent `A` has a key pair `(pk(A),sk(A))`.

The protocol has two roles: the intiator role `I` and the responder role `R`. We also add a single line comment, starting with `//`.

```
// The protocol description

protocol ns3(I,R)
```

```
{
```

Scyther works with a role-based description of the protocols. We first model the initiator role. This role has two values that are local to the role: the nonce that is created by I and the nonce that is received. We have to declare them both.

```
role I
{
  fresh ni: Nonce;
  var nr: Nonce;
```

We now model the communication behaviour of the protocol. Needham-Schroeder has three messages, and the initiator role sends the first and last of these. Note the labels (e. g., `_1`) at the end of the `send` and `recv` keywords: these serve merely to retain the information of the connected arrows in the message sequence chart.

```
  send_1(I,R, {I,ni}pk(R) );
  recv_2(R,I, {ni,nr}pk(I) );
  send_3(I,R, {nr}pk(R) );
```

Finally, we add the security requirements of the protocol. Without such claims, Scyther does not know[1] what needs to be checked.

Here we have chosen to check for secrecy of the generated and received nonce, and will check for non-injective agreement and synchronisation.

```
  claim_i1(I,Secret,ni);
  claim_i2(I,Secret,nr);
  claim_i3(I,Niagree);
  claim_i4(I,Nisynch);
}
```

This completes the specification of the initiator role.

For this simple protocol, the responder role is very similar to the initiator role[2]. In fact, there are only a few differences:

1. The keywords `var` and `fresh` have swapped places: `ni` was created by `I` and a freshly generated value there, but for the role `R` it is the received value and thus a variable.
2. The keywords `send` and `recv` have swapped places.
3. The claims should have unique labels, so they have changed, and the role executing the claim is now `R` instead of `I`.

The complete role description for the responder looks like this:

```
role R
{
  var ni: Nonce;
  fresh nr: Nonce;

  recv_1(I,R, {I,ni}pk(R) );
  send_2(R,I, {ni,nr}pk(I) );
```

[1] If you are unsure about the claims, you can also use the `--auto-claims` switch to automatically generate these at run-time.

[2] In general, the transformation is not that simple, but for many protocols this will suffice.

```
    recv_3(I,R, {nr}pk(R) );

    claim_r1(R,Secret,ni);
    claim_r2(R,Secret,nr);
    claim_r3(R,Niagree);
    claim_r4(R,Nisynch);
  }
}
```

The full protocol description file for the *Needham-Schroeder* protocol is given in Appendix A.

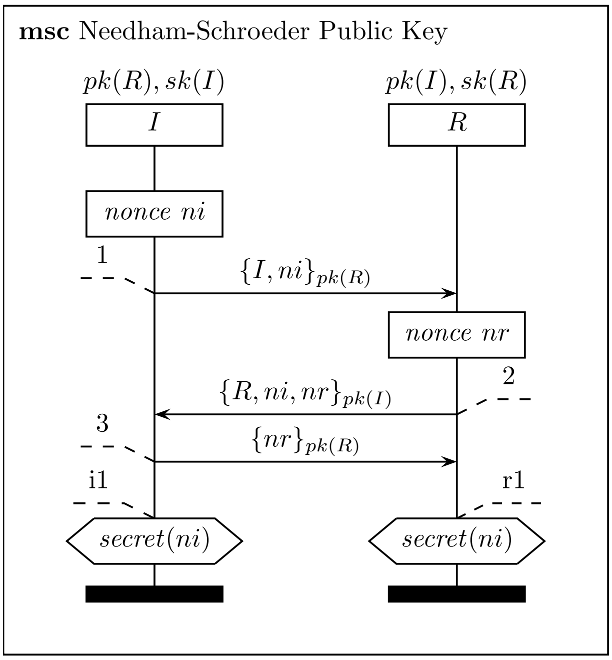

1

Figure 6.1: A message sequence chart description

Chapter 7

Specifying security properties

7.1 Specifying secrecy

7.2 Specifying authentication properties

7.2.1 Aliveness

7.2.2 Non-injective synchronisation

7.2.3 Non-injective agreement

7.2.4 Agreement over data

In order to specify data agreement, e. g., that the role I agrees with the role R on a set of terms, e. g., the nonces ni and nr, one inserts two claims:

1. At the end of the I role, insert `claim(I,Commit,R,ni,nr);`
2. In the R, just before the last send (in case of a protocol with multiple roles: the last send that causally precedes the claim in the I role), insert `claim(R,Running,I,ni,nr);`

For an example of the use of these claims, see the "ns3.spdl" input file in the Scyther distribution. For a formal definition of the signals, see [4].

Chapter 8

Using the Scyther tool GUI

The Scyther tool can be used in two main ways. First, through the graphical user interface (GUI) and second, through the command-line interface. For most users the first option is preferred.

In this section we detail the Scyther output when used through the GUI.

8.1 Results

As shown before, verifying the Needham-Schroeder public key protocol yields the following results as in Figure 8.1.

The interpretation is as follows: all the claims of the initiator role `ns3,I` are correct for an unbounded number of runs.

Unfortunately, all the claims of the responder role are false. Scyther reports that it found at least one attack for each of those four claims. We could choose to view these attacks: this will be shown in Section 8.3.

In the result window, Scyther will output a single line for each claim. The line is divided into several columns. The first column shows the protocol in which the claim occurs, and the second shows the role. In the third column a unique claim identifier is shown, of the form `p,l`, where `p` is the protocol and `l` is the claim label.[1]. The fourth column displays the claim type and the claim parameter.

Under the header `Status` we find two columns. The fifth column gives the actual result of the verification process: it will yield `Fail` when the claim is false, and `Ok` when the claim is correct. The sixth column refines the previous statement: in some cases, the Scyther verification process is not complete (which will be explored in more detail in the next section). If this column states `Verified`, then the claim is provably true. If the column states `Falsified`, then the claim is provably false. If the column is empty, then the statement of fail/ok depends on the specific bounds setting.

The seventh column, `Comments`, serves to explain the status of the results further. In particular, the column contains a single sentences. We describe the possible results below.

- `At least X attack(s)`

  Some attacks were found in the state space: however, due to the undecidability of the problem, or because of the branch and bround structure of the search, we cannot be sure that there are no other attack states.

[1] This includes the protocol name, which is important when doing multi-protocol analysis.

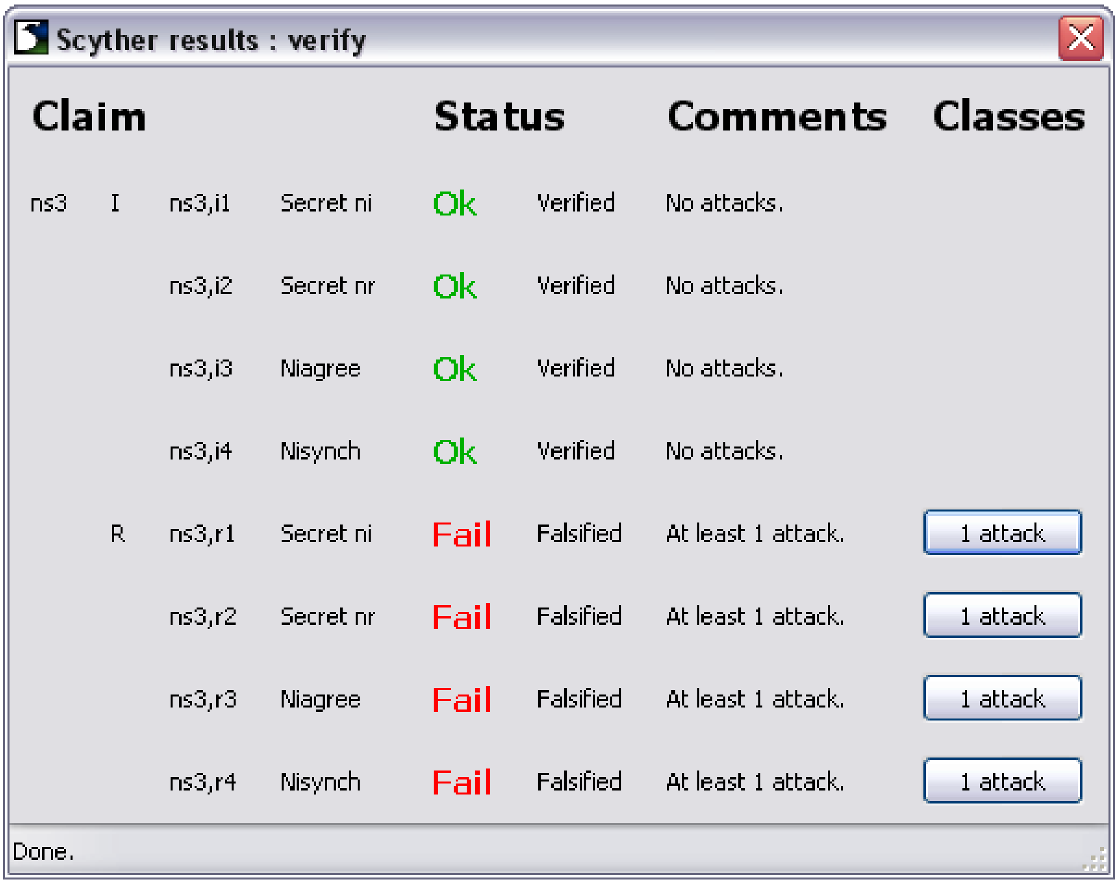

Figure 8.1: Scyther results for the Needham-Schroeder protocol

In the default setup, Scyther will stop the verification process after an attack is found.

- `Exactly X attack(s)`

  Within the statespace, there are exactly this many attacks, and no others.

- `At least X pattern(s)`

- `Exactly X pattern(s)`

  These correspond exactly to the previous two, but occur in case of a '`Reachable`' claim. Thus, the states that are found are not really attacks but classes of reachable states.

- `No attacks within bounds`

  No attack was found within the bounded statespace, but there can possibly be an attack outside the bounded statespace.

- `No attacks`

  No attack was found within the (bounded or unbounded) statespace, and a proof can be constructed that there is no attack even when the statespace is unbounded. Thus, the security property has been succesfully verified.

Note that because of the nature of the algorithm, this result can even be obtained when the statespace is bounded.

8.2 Bounding the statespace

During the verification process, the Scyther tool explores a proof tree that covers all possible protocol behaviours. The default setting is to *bound* the size of this tree in some way, ensuring that the verification procedure terminates. However, importantly, even if the size of this proof tree is bounded, unbounded verification may still be achieved.

In most cases, the verification procedure will terminate and return results before ever reaching the bound. However, if the verification procedure reaches the bound, this is reported in the result window, e. g.:

```
No attack within bounds
```

This should be interpreted as: Scyther did not find any attacks, but because it reached the bound, it did not explore the full tree, and it is possible that there are still attacks on the protocol.

The default way of bounding the *maximum number of runs*, or protocol instances. This can be changed in the `Settings` tab of the main window. If the maximum number of runs is, e. g., 5, and Scyther reports `No attack within bounds`, this means that there exist no attacks that involve 5 runs or less. However, there might exist attacks that involve 6 runs or more.

For some protocols, increasing the maximum number of runs can lead to complete results (i.e. finding an attack or being sure that there is no attack), but for other protocols the result will always be `No attack within bounds`.

Note that the verification time usually grows exponentially with respect to the maximum number of runs.

8.3 Attack graphs

In Figure 8.3 we show an attack window in more detail.

The basic elements are arrows and several kinds of boxes. The arrows in the graph represent ordering constraints (caused by the prefix-closedness of events in the protocol roles, or by dependencies in the intruder knowledge). The boxes represent creation of a run, communication events of a run, and claim events.

8.3.1 Runs

Each vertical axis represents a run (an instance of a protocol role). Thus, in this attack we see that there are two runs involved. Each run starts with a diamond shaped box. This represents the creation of a run, and is used to give information about the run.

For the run on the left-hand side in the attack we have this information:

```
Run #1
Agent2 in role I
I -> Agent2
R -> Agent1
```

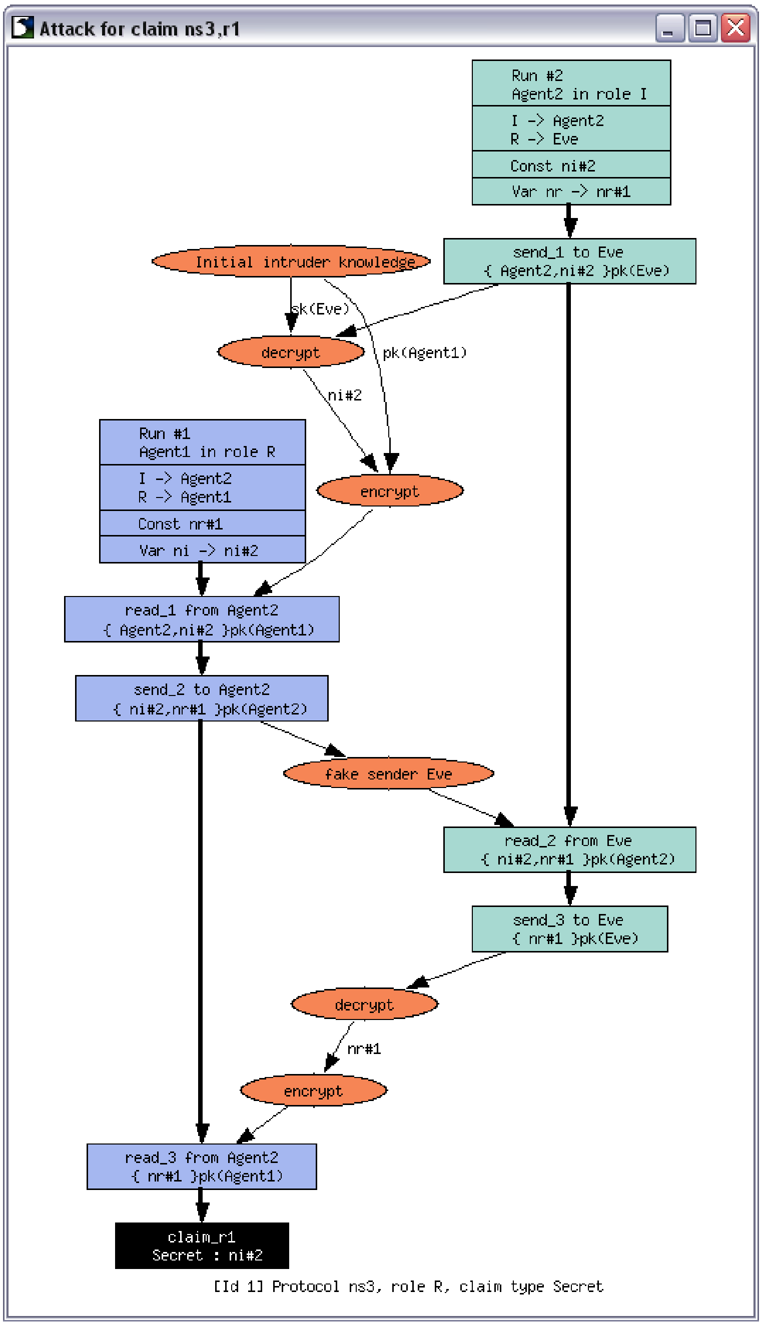

Figure 8.2: Scyther attack window

Each run is assigned a run identifier (here 1), which is an arbitrary number that enables us to uniquely identify each run. This run executes the `R` role of the protocol. It is being executed by an agent called `Agent1`, who thinks he is talking to `Agent2`. Note that although run 2 is being

executed by `Agent2`, this agent does not believe he is talking to `Agent1`.

```
Run #2
Agent2 in role I
I -> Agent2
R -> Eve
```

In the run on the right, we see This run represents an instance of the role `I`. From the second line we can see which agent is executing the run, and who he thinks he is talking to. In this example, the run is executed by an agent called `Agent2`, who thinks the responder role is being executed by the untrusted agent `Eve`.[2]

Additionally, the run headers contain information on the freshly generated values (e. g., run 1 generates `nr#1`) and information on the instantiation of the local variables (e. g., run 1 instantiates its variable `ni` with the nonce `ni#2` or run 2.

8.3.2 Communication events

Send events denote the sending of a message. The first send occurs in this attack is the first send event of run 2.

```
send_1(Eve, { Agent#0, ni#2 }pk(Eve) )
```

Every time a message is sent, it is effectively given to the intruder. In this case, because the intruder knows the secret key `sk(Eve)` of the agent `Eve`, he can decrypt the message and learns the value of the nonce `ni#2`.

Receive events correspond to the successful reception of a message. The first receive event that can occur in this attack is the first receive event of run 0.

```
recv_1(Agent#0, { Agent#0, ni#2 }pk(Agent#1) )
```

This tells us that the agent executing this run, `Agent#1`, reads a message that is apparently coming from `Agent#1`. The message that is received is { `Agent#0, ni#2` }`pk(Agent#1)` : the name of the agent he thinks he is communicating with and the nonce `ni#2`, encrypted with his public key.

The incoming arrow does not indicate a direct sending of the message. Rather, it denotes an ordering constraint: this message can only be received *after* something else has happened. In this case, we see that the message can only be received after run 2 sends his initial message. The reason for this is the nonce `ni#2`: the intruder cannot predict this nonce, and thus has to wait until run 2 has generated it.

In the graph the connecting arrow is red and has a label "construct" with it: this is caused by the fact that the message sent does not correspond to the message that is received. We know the intruder can only construct the message to be received after the sent message, and thus it must be the case that he uses information from the sent message to construct the message that is received. Other possibilities include a green and a yellow arrow. A yellow arrow indicates that a message was sent, and received in exactly the same form: however, the agents disagree about who was sending a message to whom. It is therefore labeled with "redirect" because the intruder

[2]Because this agent is talking to the untrusted agent, of course all information is leaked, and no guarantees can be given.

must have redirected the message. A green arrow (not in the picture) indicating that a message is received exactly the same as it was sent, representing a normal message communication between two agents.

Note that a `recv` event without an incoming arrow denotes that a term is received that can be generated from the initial knowledge of the intruder. There is no such event in the example, but this can occur often. For example, if a role reads a plain message containing only an agent name, the intruder can generate the term from his initial knowledge.

8.3.3 Claims

Chapter 9

Using the Scyther command-line tools

All of the features offered by the Scyther GUI are also available through command-line tools. Additionally, the command-line tools offer some features that currently cannot be accessed through the GUI.

Depending on your platform, the Scyther directory contains one of the following executables:

- `Scyther/scyther-linux`
- `Scyther/scyther-w32`
- `Scyther/scyther-mac`

In the following, we assume that the linux version is used. If you have a different version, please replace `scyther-linux` in the below by the executable for your platform.

To get a list of (some) of the command-line options, run the executable with the `--help` switch, e.g.:

```
scyther-linux --help
```

To analyze the Needham-Schroeder protocol and generate a .dot file (the input language for the Graphviz tool) for the attacks, use

```
scyther-linux --dot-output --output=ns3-attacks.dot ns3.spdl
```

The resulting output file can then be rendered by graphviz, e.g.:

```
dot -Tpdf -O ns3-attacks.dot
```

This yields several PDF files `ns3-attacks.dot[.N].pdf` that contain the attack graphs.

To get a more complete list of command-line options, run the executable with the `--expert --help` switch, e.g.:

```
scyther-linux --expert --help
```

Chapter 10

Advanced topics

10.1 Modeling more than one asymmetric key pair

Asymmetric keys are typically modeled as two functions: one function that maps the agents to their public keys, and another function that maps agents to their secret keys.

By default, each agent x has a public/private key pair $(\texttt{pk}(\texttt{x}), \texttt{sk}(\texttt{x}))$.

To model other asymmetric keys, we first define the two functions, which are for example named `pk2` for the public key function, and `sk2` for the secret key function.

```
const pk2: Function;
secret sk2: Function;
```

We also declare that these functions represent asymmetric key pairs:

```
inversekeys (pk2,sk2);
```

If defined in this way, a term encrypted with `pk2(x)` can only be decrypted with `sk2(x)` and vice versa.

10.2 Approximating equational theories

The operational semantics underlying Scyther currently only consider syntactic equality: two (ground) terms are equal if and only if they are syntactically equivalent. However, there are several common cryptographic constructions that are more naturally modeled by using certain equalities. For example:

1. $g^{ab} \pmod N$ and $g^{ba} \pmod N$, to model Diffie-Hellman exponentiation.
2. $k(A, B)$ and $k(B, A)$, to model bidirectional long-term keys.

Although Scyther does not provide direct support for such equational theories, there exists a straightforward underapproximation.

The core idea is that instead of modeling the term equality, we provide the adversary with the ability to learn all terms in an equivalence class if he learns one of its elements. For example, for the equivalence class $\{k(A, B), k(B, A)\}$ we can provide the adversary with the ability to learn $k(B, A)$ from $k(A, B)$, and vice versa. We can model this by introducing an appropriate helper protocol (denoted by the prefix '@'):

```
protocol @keysymmNaive(X) {
  role X {
    var Y: Agent;

    recv_!1(X,X, k(X,Y) );
    send_!2(X,X, k(Y,X) );
  }
}
```

Because the role can be instantiated for any agents X and Y, this covers all possible combinations of agents.

The above naive approximation can be significantly improved. One obvious and practically relevant omission is that the adversary usually learns encrypted messages, but not the key. In such cases, we still would like to model that `{ m }k(A,B)` = `{ m }k(B,A)`. Thus we adapt our helper protocol:

```
protocol @keysymmInefficient(X,Y) {
  role X {
    var Y: Agent;

    recv_!1(X,X, k(X,Y) );
    send_!2(X,X, k(Y,X) );
  }
  role Y {
    var X: Agent;
    var m: Ticket;

    recv_!1(Y,Y, { m }k(X,Y) );
    send_!2(Y,Y, { m }k(Y,X) );
  }
}
```

If the protocol contains further terms in which the symmetric keys appear in other positions, such as in nested encryptions or hashes, we would add further roles.

The above approximation is often inefficient in practice. We can improve performance by making the helper protocol rules more tight, i. e., by exploiting more type information about the protocol. For example, if the protocol transmits two types of encrypted messages:

1. `{ I,nI,nR }k(I,R)` , and
2. `{ nI }k(I,R)` ,

then we would modify the helper protocol in the following way:

```
protocol @keysymm(X,Y,Z) {
  role X {
    var Y: Agent;

    recv_!1(X,X, k(X,Y) );
    send_!2(X,X, k(Y,X) );
  }
  role Y {
    var X,Z: Agent;
    var n1,n2: Nonce;
```

```
    recv_!1(Y,Y, { Z,n1,n2 }k(X,Y) );
    send_!2(Y,Y, { Z,n1,n2 }k(Y,X) );
  }
  role Z {
    var X,Y: Agent;
    var n1: Nonce;

    recv_!1(Z,Z, { n1 }k(X,Y) );
    send_!2(Z,Z, { n1 }k(Y,X) );
  }
}
```

In general, one would manually inspect the protocol and extract all positions in which a term from an equivalence class occurs as a subterm. For each of these positions, we model an appropriate role in the helper protocols.

This is also used to model, for example, Diffie-Hellman exponentiation. For exponentiation we introduce an abstract function symbol, e. g., `exp`, and a public constant `g`. We then introduce a helper protocol with roles to model that `exp(exp(g,X),Y)` = `exp(exp(g,Y),X)`.

In practice, this type of underapproximation has proven to be extremely effective, to the point that all known attacks on real-world protocols that can be modeled using the "real" equational theory, are found by Scyther when using the underapproximation.

One caveat is that while this approximation works well for secrecy and data-agreement, it can cause message-based agreement properties (such as synchronisation) to fail, because their message equality checks are syntactical. These checks are not affected by the introduction of helper protocols.

10.3 Modeling time-stamps and global counters

Scyther's underlying protocol model currently does not provide support for variables that are shared among the runs of an agent. Effectively, each run starts with a "clean slate", independent of any runs that have been executed previously. In other words, globally update state can not be modeled directly.

In the following sections we provide some modeling approaches for common problems.

10.3.1 Modeling global counters

Globally incremented counters can be modeled using freshly generated values. This ensures that each run uses a different value. The model is coarse in the sense that the recipient of such a counter cannot check that it is the successor of the previous value of the counter.

10.3.2 Modeling time-stamps using nonces

There are at least two ways to model time-stamps.

The first model is more appropriate for protocols where the probability that a given time-stamp value is accepted by two runs is very low. This occurs when time-stamps have great precision or when two runs occur only sequentially, possibly with some delay time in between. In this case, one can model time-stamps as freshly generated values, e. g., nonces. To cater for the fact that the adversary typically knows the time (and thus can also predict time-stamps), we prepend a send event to the role that provides the adversary with the value of the time-stamp that will

be used. For example, we would prepend the send with label `!T1` for time-stamp `T1` as in the following example:

```
usertype Timestamp;

protocol MyProtocol(Server,Client) {
  role Server{
    fresh T1: Timestamp;

    /* Time-stamps are unique per run */
    send_!T1(Server, Server, T1);

    ...
    /* Server uses time-stamp value */
    send_2(Server,Client, { Server, T1 }pk(Client) );
    ...
  }
}
```

10.3.3 Modeling time-stamps using variables

The second model is more appropriate when it is reasonable that two runs may accept the same time-stamp value. This is common for coarse time-stamps, or for roles that are typically executed with high parallelism, such as server roles. In such cases, one can instead model timestamps as values that are determined by the adversary. In contrast to the previous solution, this is done by prepending a receive event. For example:

```
usertype Timestamp;

protocol MyProtocol(Server,Client) {
  role Server{
    var T1: Timestamp;

    /* Adversary chooses time-stamp value */
    recv_!T1(Server, Server, T1);

    ...
    /* Server uses time-stamp value */
    send_2(Server,Client, { Server, T1 }pk(Client) );
    ...
  }
}
```

10.4 Multi-protocol attacks

Scyther can be used to check for so-called *multi-protocol attacks* (closely related concepts are *cross-protocol attacks* and *chosen protocol attacks*). These attacks depend on the interactions between different (sub)protocols: sometimes the adversary can use messages or message components from one protocol to attack another. For more information on this type of attack we refer to [2, 3].

The easiest way to check for multi-protocol attacks in Scyther is to combine two protocol descriptions into a single file, i.e., create a new `.spdl` file and paste into this file two other `.spdl`

files. The resulting file models an environment in which both protocols are running. Use Scyther to evaluate the claims in the combined file.

Chapter 11

Further reading

Bibliography

[1] Cas Cremers and Sjouke Mauw. *Operational Semantics and Verification of Security Protocols*. Information Security and Cryptography. Springer, 2012.

[2] C.J.F. Cremers. Feasibility of multi-protocol attacks. In *Proc. of The First International Conference on Availability, Reliability and Security (ARES)*, pages 287–294, Vienna, Austria, April 2006. IEEE Computer Society.

[3] J. Kelsey, B. Schneier, and D. Wagner. Protocol interactions and the chosen protocol attack. In B. Christianson, B. Crispo, T.M.A. Lomas, and M. Roe, editors, *Fifth International Workshop on Security Protocols*, volume 1361 of *LNCS*, pages 91–104. Springer, 1997.

[4] G. Lowe. A hierarchy of authentication specifications. In *Proc. 10th IEEE Computer Security Foundations Workshop (CSFW)*, pages 31–44. IEEE, 1997.

Appendix A

Full specification for Needham-Schroeder public key

```
/*
 * Needham-Schroeder protocol
 */


// The protocol description

protocol ns3(I,R)
{
  role I
  {
    fresh ni: Nonce;
    var nr: Nonce;

    send_1(I,R, {I,ni}pk(R) );
    recv_2(R,I, {ni,nr}pk(I) );
    claim(I,Running,R,ni,nr);
    send_3(I,R, {nr}pk(R) );

    claim_i1(I,Secret,ni);
    claim_i2(I,Secret,nr);
    claim_i3(I,Alive);
    claim_i4(I,Weakagree);
    claim_i5(I,Commit,R,ni,nr);
    claim_i6(I,Niagree);
    claim_i7(I,Nisynch);
  }

  role R
  {
    var ni: Nonce;
    fresh nr: Nonce;

   recv_1(I,R, {I,ni}pk(R) );
```

```
    claim(R,Running,I,ni,nr);
    send_2(R,I, {ni,nr}pk(I) );
    recv_3(I,R, {nr}pk(R) );

    claim_r1(R,Secret,ni);
    claim_r2(R,Secret,nr);
    claim_r3(R,Alive);
    claim_r4(R,Weakagree);
    claim_r5(R,Commit,I,ni,nr);
    claim_r6(R,Niagree);
    claim_r7(R,Nisynch);
  }
}
```

Index

@, 20
`!`, 18

abbreviate, 21
`Agent`, 17
agreement (on messages), 19
agreement on data, 19, 29
`Alive`, 18
asymmetric key
    multiple pairs per agent, 39
asymmetric keys, 17
at least X attack(s), 31
at least X pattern(s), 32
atomic term, 15
attack graph, 33
attack window, 33

bidirectional keys, 39
BNF, 23

case-sensitive, 15
chosen protocol attacks, 42
`claim`, 18
claim event, 18
command-line tools, 37
comments, 15
`Commit`, 19, 29
communication event, 35
`const`, 21
constant, 16
construct, 35
counter, *see* global counter
cross-protocol attacks, 42

data agreement, 19, 29
define macro, 21
Diffie-Hellman exponentiation, 39
downloading Scyther, 9

`Empty`, 19
equational theories, 39
event
    claim, 18
    match, 19
    not match, 19
    recv, 18
    send, 18
events, 18
exactly X attack(s), 32
exactly X pattern(s), 32
exponentiation, *see* Diffie-Hellman exponentiation

freshly generated value, 16
`Function`, 17

global constant, 16
global counter, 41
global declarations, 21
GUI, 31
    using Scyther without GUI, 37

hash functions, 17
`hashfunction`, 17
helper protocol, **20**, 39

identifier, 15
import file, 22
`include`, 22
input file, 22
installing Scyther, 9
internal computation events, 19

`k(X,Y)`, 16

`macro`, 21
match event, 19
message agreement, 19
multi-protocol attacks, 42
multiple asymmetric key pairs per agent, 39
multiple roles per agent, 23

Needham-Schroeder protocol, 25

`Niagree`, 19
`Nisynch`, 19
no attacks, 32
no attacks within bounds, 32
non-injective agreement, 19, 29
non-injective synchronisation, 19
`Nonce`, 17
nonce, 16
not match event, 19
NS, *see* Needham-Schroeder protocol

one role per agent, 23
`one-role-per-agent`, 23

pairing, 16
pattern match events, 19
`pk(X)`, 17
protocol definition, 20

quick start tutorial, 11

random value, 16
`Reachable`, 19
`read`, 18
`recv`, 18
role definition, 20
run, 33
`Running`, 19, 29

Scyther website, 5
`Secret`, 18
security properties, 18
`send`, 18
`sk(X)`, 17
`SKR`, 18
symmetric keys, 16
symmetric-role protocol, **21**
synchronisaton, 19
syntactic equality, 39

`Ticket`, 17
time-stamps, 41–42
tupling, 16

`usertype`, 17

var, 16
variable, 16
verification, 32

`Weakagree`, 18
website, *see* Scyther website
whitespace, 15
